# Indole bearing thiadiazole analogs: synthesis, β-glucuronidase inhibition and molecular docking study

**DOI:** 10.1186/s13065-019-0522-x

**Published:** 2019-02-04

**Authors:** Noor Barak Almandil, Muhammad Taha, Mohammed Gollapalli, Fazal Rahim, Mohamed Ibrahim, Ashik Mosaddik, El Hassane Anouar

**Affiliations:** 1 Department of Clinical Pharmacy, Institute for Research and Medical Consultations (IRMC), Imam Abdulrahman Bin Faisal University, P. O. Box 1982, Dammam, 31441 Saudi Arabia; 2 Department of Chemistry, Hazara University, Mansehra, 21300 Khyber Pakhtunkhwa Pakistan; 3 Department of Computer Information Systems, College of Computer Science & Information Technology, Imam Abdulrahman Bin Faisal University, P. O. Box 1982, Dammam, 31441 Saudi Arabia; 4 Department of Chemistry, College of Sciences and Humanities, Prince Sattam Bin Abdulaziz University, P.O. Box 83, Al-Kharj, 11942 Saudi Arabia

**Keywords:** Synthesis, Indole, Thiadiazole, β-Glucuronidases, Molecular docking, SAR

## Abstract

Indole based thiadiazole derivatives (**1**–**22**) have synthesized, characterized by NMR and HREI-MS and evaluated for β-Glucuronidase inhibition. All compounds showed outstanding β-glucuronidase activity with IC_50_ values ranging between 0.5 ± 0.08 to 38.9 ± 0.8 µM when compared with standard d-saccharic acid 1,4 lactone (IC_50_ value of 48.1 ± 1.2 µM). The compound **6**, a 2,3-dihydroxy analog was found the most potent among the series with IC_50_ value 0.5 ± 0.08 µM. Structure activity relationship has been established for all compounds. To confirm the binding interactions of these newly synthesized compounds, molecular docking study have been carried out which reveal that these compounds established stronger hydrogen bonding networks with active site residues.

**Electronic supplementary material:**

The online version of this article (10.1186/s13065-019-0522-x) contains supplementary material, which is available to authorized users.

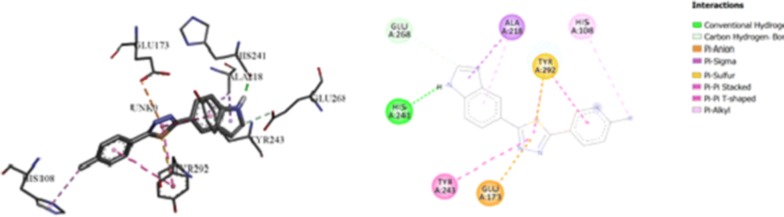

## Background

β-Glucuronidases enzymes belong to family glycoside hydrolase GH1, GH2 and GH79, and reduce glucuronic acid sugar moiety from non-reducing termini. It has been used in disease diagnosis, gene manipulation and food industry. Recently it has drawn more attention to enhance the efficacy by modifying natural glucuronides [1–5]. β-Glucuronidase present in microsomes and lysosomes and eliminate from body through urinary track [6]. Activity of β-glucuronidase increases in many diseases like AIDS, inflammation, cancer and hepatic disease [7]. Cholelithiasis is originated in human bile due to endogenous biliary β-glucuronidase which is related with deconjugation of bilirubin glucuronidase [8]. Increase level of this enzyme is associated with some urinary disorder like active pyelonephritis, cancer of kidney bladder and acute renal necrosis [9]. β-Glucuronidase in humane has resulted in mucopolysaccharidosis type VII (MPS VII; Sly syndrome), which is characterized by growth of glycosaminoglycans in cells of most tissues [10, 11]. Indole is an important class of compounds with wide range of application in medicinal chemistry [12, 13]. Variety of compounds having indole is the basic unit possesses antitumor applications [14, 15]. Many compounds containing indole scaffold have effect in many physiological processes. Indole with 5-HT receptor activity as agonist and antagonist is the most recent synthetic interest in medicinal chemistry [16, 17].

The thiadiazole skeleton constitutes an important central template for a wide variety of biologically active compounds, having many pharmacological applications 2-amino-1,3,4-thiadiazole and certain structurally related compounds have been known for 50 years to have antitumor activity [18]. Compounds of this class are uricogenic agents in man [19]. Both the antitumor and the uricogenic activities can be prevented or reversed by nicotinamide [20, 21]. Variety of thiadiazole derivatives possess interesting biological activities and are of great interest to chemist [22]. Many bioactive molecules in the field of drugs and pharmaceuticals contained thiadiazole moiety [23–25]. Thiadiazole derivatives biological applications include antibacterial, anticonvulsant, anti-leishmanial, anticancer, antidepressant, anti-inflammatory, anti-oxidant and anti-tuberculosis [26–34]. The indole and related heterocyclic compounds have also great importance in the field of chemistry [35].

Keeping in view the great biological potential of indole and thiadiazole analogs here in this study we have planned to synthesize the hybrid molecules of indole based thiadiazole derivatives with the hope that it may showed greater potential. After evaluation for biological potential we have found outstanding results which support our previous hypothesis. Here we are reporting synthesis of indole based thiadiazole derivatives, its characterization, β-glucuronidase inhibition and molecular docking studies.

## Result and discussion

### Chemistry

**Scheme 1:**
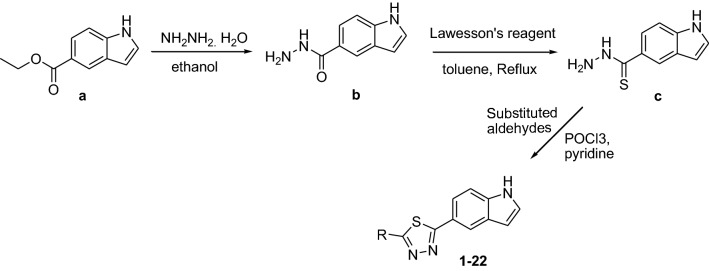
Synthesis of indole based thiadiazole derivatives **1**–**22**

A series of indole based thiadiazole were synthesized by refluxing ethyl 1*H*-indole-5-carboxylate (**a**) with hydrazine hydrate in ethanol for 2 h to afford 1*H*-indole-5-carbohydrazide (**b**) refluxed with Lawesson’s reagent in toluene yielded corresponding thio-analogue (**c**). Thiohydrazide (**c**) was then treated with various aryl aldehydes to form cyclized adducts **1**–**22** (Table 1) in the presence of POCl_3_ (Scheme 1). Upon completion of reaction (monitored with TLC), product was recrystallized from methanol and purified by washing. Spectral data including ^1^H-NMR, ^13^C-NMR, and HREI-MS for all synthesized compounds were recorded.

**Table 1:**
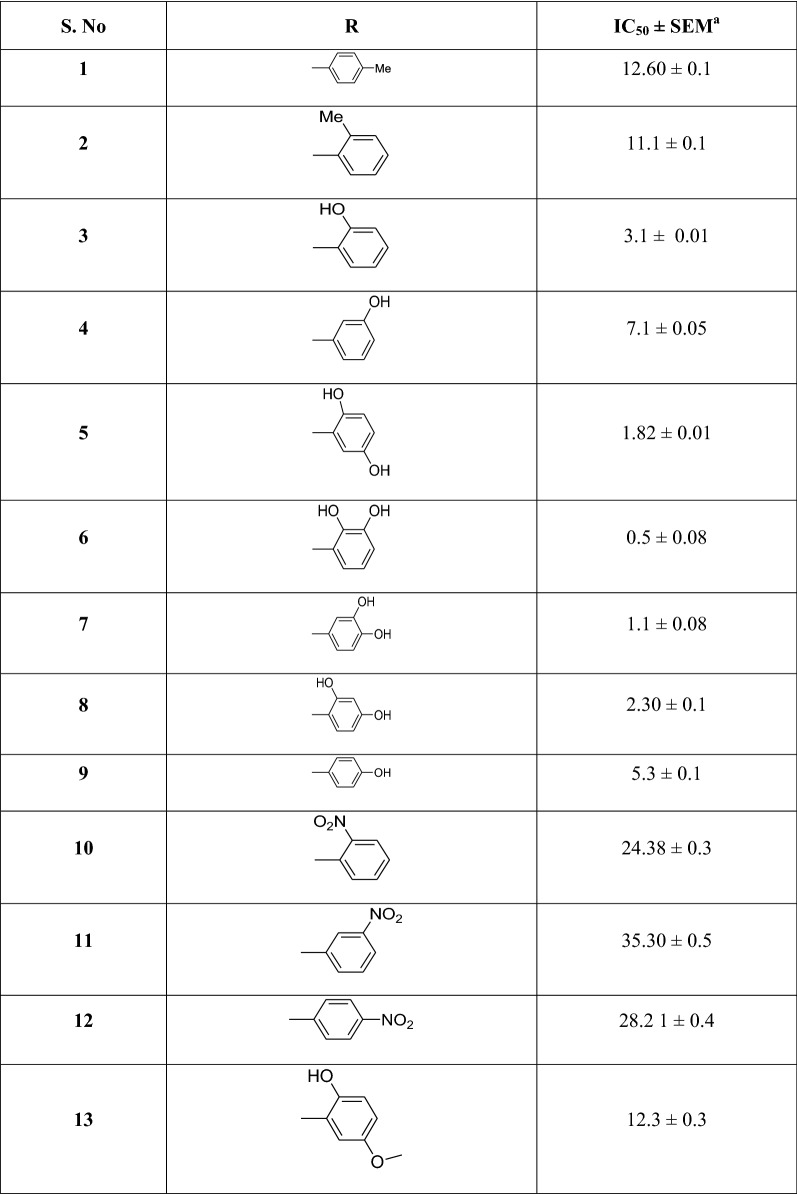

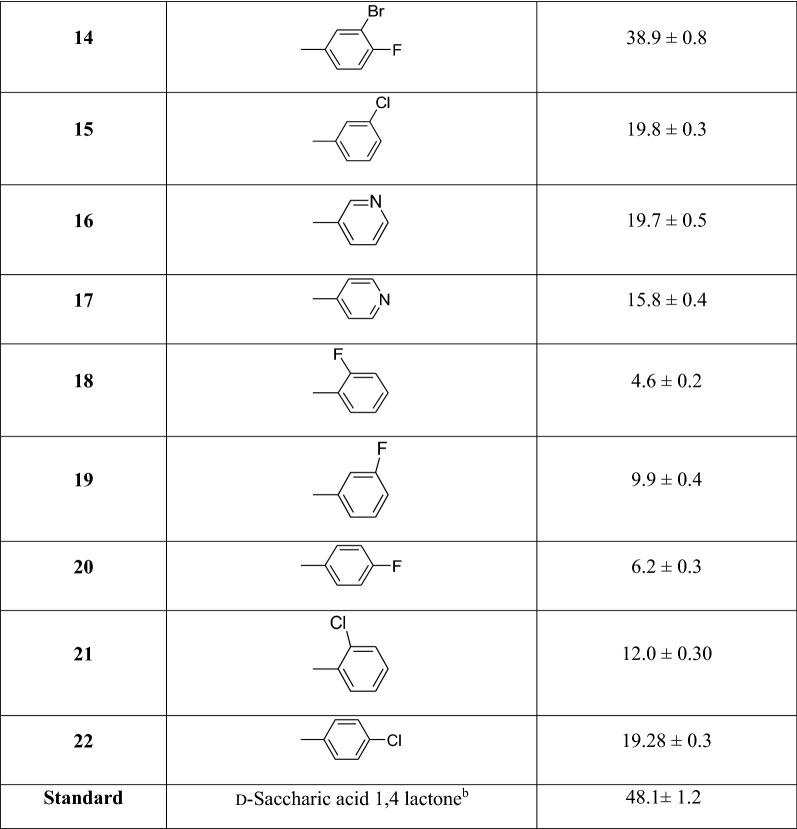
Synthesis of indole based thiadiazole analogs and their β-glucuronidase potential

^a^standard error mean

^b^standard drug

### Biological activity

In the continuation of our effort for enzyme inhibition [36], we have synthesized series of indole based thiadiazole derivatives, a new class of β-glucuronidase inhibitors. All the compounds (**1**–**22**) were screened for β-glucuronidase activity. All the compounds showed outstanding inhibition when compared with standard d-saccharic acid 1,4 lactone with IC_50_ value 48.1 ± 1.2 µM (Table 1). Structure activity relationship (SAR) has been established mainly based on the substitution pattern on phenyl ring attached to thiadiazole. The compound **6**, a 2,3-dihydroxy analog was found the most potent among the series with IC_50_ value 0.5 ± 0.08 µM. The greater potential shown by this compound is seems mainly due to the hydroxyl group on phenyl ring which might be involve in hydrogen bonding with the active site of enzyme. If we compare analog **6** with other dihydroxy substituted analogs like **5**, a 2,5-dihydroxy analog (IC_50_ = 1.82 ± 0.01 µM), **7**, a 3,4-dihydroxy analog (IC_50_ = 1.1 ± 0.08 µM), and **8**, a 2,4-dihydroxy analog (IC_50_ = 2.30 ± 0.1 µM), the analog **6** is superior. The slight difference in the potential of these analogs is seems due to the difference in position of substituent. The mono-hydroxy analogs like **3**, a 2-hydroxy analog (IC_50_ = 3.1 ± 0.01 µM), **4**, a 3-hydroxy analog (IC_50_ = 7.1 ± 0.05 µM), **9**, a 4-hydroxy analog (IC_50_ = 5.3 ± 0.1 µM) and **13**, a 2-hydroxy-4-methoxy analog (IC_50_ = 12.3 ± 0.3 µM) also showed outstanding potential. The dihydroxy analogs are superior when mono-hydroxy analogs. The reason for greater potential of dihydroxy analogs is mainly due to greater number of hydroxyl group. This shows that number of hydroxyl group also paly critical role in this inhibition. In nitro substituted analogs like **10**, a 2-nitro analog (IC_50_ value 24.38 ± 0.3 µM) is predominating over **11**, a 3-nitro analog (IC_50_ value 35.30 ± 0.5 µM) and **12**, a 4-nitro analog (IC_50_ value 28.2 1 ± 0.4 µM) which shows that position of substituent plays an important role. Similar pattern was also observed in other substituted analog like flouro analogs **18**, a 2-nitro analog (IC_50_ value 4.6 ± 0.2 µM) with **19**, a 3-nitro analog (IC_50_ value 9.9 ± 0.4 µM) and **20**, a 4-nitro analog (IC_50_ value 6.2 ± 0.3 µM) and chloro substituted analogs like compound **21**, a 4-chloro analog (12.0 ± 0.30 µM) is more potent than **22**, a 4-chloro analog (IC_50_ value 19.28 ± 0.3 µM)and **15**, a 3-chloro analog (IC_50_ value 19.8 ± 0.3 µM). It was concluded form this study that position, nature and number of substituents on phenyl ring plays a critical in this inhibition.

### Molecular docking study

The concentration inhibition IC_50_ values of thiadiazole synthesized derivatives as β-glucoronidase inhibitors are presented in (Table 1). As shown in Table 1, the inhibitory potency of the tilted compounds depends mainly on the type, number and positions of the functional group in the substitute group R of the synthesized derivatives. According to inhibitory IC_50_ values (Table 1), the synthesized derivatives may be subdivided into highly active group with low IC_50_ values (e.g., **6**, **7**, **3**), moderate active group (e.g., **4**, **9**, **20**) and low active group (e.g., **1**, **2**). For a better understanding of the observed results and to rationalize the highest activity of **6** compared to **4**, and the low activity of **1** with regards to **4**, molecular docking study has been carried out to shed light on the established binding modes of the three chosen synthesized compounds **6**, **4** and **1** to the closest residues in the active site of β-glucoronidase enzyme. Table 2 summarized the calculated binding energies of the stable complexes ligand- β-glucoronidase, number of established intermolecular hydrogen bonding between the synthesized compounds (**1**, **4** and **6**) and active site residues of β-glucoronidase.

**Table 2:** Concentration inhibition IC_50_, docking binding energies and number of closest residues to the docked ligand in the active site of synthesized derivatives (**1**, **4** and **6**) within the active binding site of β-glucoronidase

No. of compound	Free binding energy (kcal/mol)	H-bonds (HBs)	Number of closest residues to the docked ligand in the active site	IC_50_ ± SEM
1	− 7.71	1	7	12.60 ± 0.1
4	− 8.29	2	8	7.1 ± 0.05
6	− 8.59	4	7	0.5 ± 0.08

**Fig. 1:**
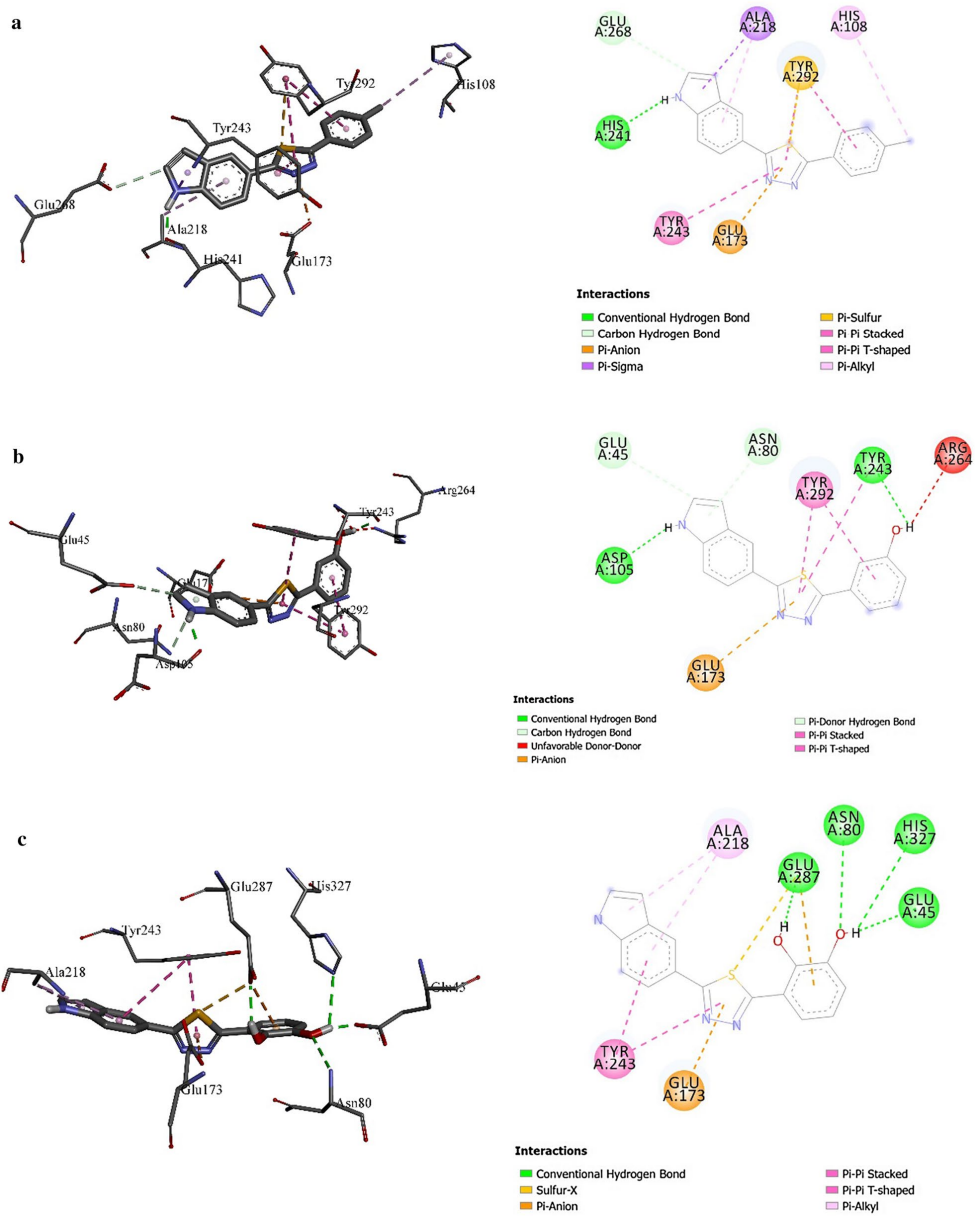
3D (right) and 2D (left) closest interactions between active site residues of β-glucuronidase and synthesized compounds **a 1**, **b 4**, and **c 6**

As can be seen from the docking results shown in Table 2 and Fig. 1, the highest activity of synthesized compound **6** compared to low active compounds **4** and **1** is mainly return to (ii) the stability of the formed complex between the docked compounds and β-glucoronidase, and the (ii) number of hydrogen bonding established between the docked ligands and the active site residues of the β-glucoronidase. However, it can be concluded that the number of closest residues to the docked ligands where all of them are surrounded by almost the same number of residues into the active site (Table 2 and Fig. 1) has no effect on the observed activities. Indeed, the formed complex between **6** and β-glucoronidase has the lowest binding energy of − 8.6 kcal/mol compared to **4** and **1** with binding energies of the stables complexes between the two compound and β-glucoronidase are of − 8.3 and − 7.7 kcal/mol, respectively (Table 2). In addition, four hydrogen bonding are established between residues of β-glucoronidase and compound **6** into the active site (Fig. 1c). The strongest hydrogen bond is formed between GLU45 amino acid and the hydrogen atom of hydroxyl group of catechol moiety of a **6** with distance of 1.66 Å. The second hydrogen bond is relatively weaker than the first one and is established between hydrogen atom of hydroxyl of catechol oxygen in *ortho* position of catechol group and GLU287 with a distance of 2.2 Å. The two other hydrogen bonds are relatively weak than the previous ones. The first one is established between ASN80 amino acid and the oxygen atom of the hydroxyl group of catechol moiety of **6** with a distance of 2.82 Å. The second one is established between HIS327 and hydrogen atom of hydroxyl group of catechol moiety of **6** with a distance of 2.96 Å. In a similar way, the higher activity of **4** compared to **1** may be explained by the above effects (i) and (ii) (Table 1 and Fig. 1). For instance, the complex formed between **4** and β-glucoronidase has a binding energy of − 8.3 kcal/mol and two hydrogen bonding of distance 1.95 Å, which are formed between amino acids ASP105 and TYR243 and hydrogen of NH and hydrogen atom of hydroxyl group of phenol group of **4**, respectively. While, for the synthesized compound **1**, the formed complex has energy binding of − 7.7 kcal/mol, and only one hydrogen bond that is formed between HIS241 amino acids and NH group of compound **1**.

## Materials and methods

NMR experiments were performed on Avance Bruker AM 300 MHz machine. Electron impact mass spectra (EI MS) were recorded on a Finnigan MAT-311A (Germany) mass spectrometer. Thin layer chromatography (TLC) was performed on pre-coated silica gel aluminum plates (Kieselgel 60, 254, E. Merck, Germany). Chromatograms were visualized by UV at 254 and 365 nm.

### Molecular docking details

The interaction binding modes between the active site residues of β-glucoronidase and docked synthesized indole derivatives have been carried out using Autodock package [37–39]. X-ray coordinates of β-glucoronidase and the originated docked ligand N-alkyl cyclophellitol aziridine were downloaded from the RCSB data bank web site (PDB code 5G0Q) [40–45]. Water molecules were removed; polar hydrogen atoms and Kollman charge were added to the extracted receptor structure by using the automated tool in AutoDock Tools 4.2. The active site is identified based on co-crystallized receptor-ligand complex structure of β-glucoronidase. The re-docking of the original ligand *N*-alkyl cyclophellitol aziridine into the active site is well reproduced with a RMSD value less than 2 Å. The molecular structures geometries of indole synthesized derivatives were minimized at Merck molecular force field 94 (MMFF94) level 44. The optimized structures were saved as pdb files. Nonpolar hydrogens were merged and rotatable bonds were defined for each docked ligand. Docking studies were performed by Lamarckian genetic algorithm, with 500 as total number of run for binding site for originated ligand and 100 run for the synthesized derivatives. In each respective run, a population of 150 individuals with 27,000 generations and 250,000 energy evaluations were employed. Operator weights for crossover, mutation, and elitism were set to 0.8, 0.02, and 1, respectively. The binding site was defined using a grid of 40 × 40 × 40 points each with a grid spacing of 0.375 Å. The docking calculation have been carried out using an Intel (R) Core (TM) i5-3770 CPU @ 3.40 GHz workstation (Additional file 1).

### General procedure for the synthesis of compounds *(1*–*22)*

Thiadiazole derivatives **1**–**22** were synthesized by refluxing ethyl 1*H*-indole-5-carboxylate (**a**) with hydrazine hydrate in ethanol for 2 h to afford 1*H*-indole-5-carbohydrazide (**b**) refluxed with Lawesson’s reagent in toluene yielded corresponding thio-analogue (**c**). Thiohydrazide (**c)** was then treated with various aryl aldehydes to form cyclized adducts **1**–**22**.

#### Compound **1**: *2*-*(1H*-*indol*-*5*-*yl)*-*5*-*(p*-*tolyl)*-*1,3,4*-*thiadiazole*

Yield 90%, ^1^H-NMR (500 MHz, DMSO-*d*_*6*_): *δ* 11.75 (s, 1H), 8.18 (s, 1H), 7.68 (d, 1H, *J* = 8.0 Hz), 7.60 (d, 2H, *J* = 6.5 Hz), 7.45 (t, 2H, *J* = 8.0 Hz), 7.26 (d, 2H, *J* = 7.0 Hz), 6.53 (s, 1H), 2.38 (s, 3H); ^13^C-NMR (125 MHz, DMSO-*d*_*6*_): *δ* 173.8, 173.4, 133.3, 131.4, 130.3, 130.1, 129.5, 129.4, 128.4, 127.4, 127, 124.1, 119.4, 116.2, 111.2, 102.2, 21.1, EI-MS: m/z calcd for C_17_H_13_N_3_S [M]^+^ 291.0830, Found 291.0818.

#### Compound **2:***2*-*(1H*-*indol*-*5*-*yl)*-*5*-*(o*-*tolyl)*-*1,3,4*-*thiadiazole*

Yield 87%, ^1^H-NMR (500 MHz, DMSO-*d*_*6*_): *δ 11.70 (s, 1H),* 8.23 (s, 1H), 7.80 (d, 1H, *J* = 6.0 Hz), 7.68 (d, 1H, *J *= 8.0 Hz), 7.48-7.45 (m, 2H), 7.29-7.26 (m, 3H), 6.55 (s, 1H), 2.43 (s, 3H); ^13^C-NMR (125 MHz, DMSO-*d*_*6*_): *δ* 173.8, 173.1, 137, 136.7, 135.3, 129.8, 129.4, 128.4, 128.3, 127.7, 126.3, 124.7, 119, 116.2, 111.2, 102.2, 18.5, EI-MS: m/z calcd for C_17_H_13_N_3_S [M]^+^ 291.0830, Found 291.0813.

#### Compound **3**: *2*-*(5*-*(1H*-*indol*-*5*-*yl)*-*1,3,4*-*thiadiazol*-*2*-*yl)phenol*

Yield 83%, ^1^H-NMR (500 MHz, DMSO-*d*_*6*_): *δ* 11.90 (s, 1H, NH), 8.52 (s, 1H, OH), 8.20 (s, 1H), 7.68 (d, 1H, *J* = 8.0 Hz), 7.59 (d, 1H, *J *= 8.0 Hz), 7.52 (d, 2H, *J *= 7.5 Hz), 6.50 (d, 2H, *J* = 8.0 Hz), 6.57 (s, 1H); ^13^C-NMR (125 MHz, DMSO-*d*_*6*_): *δ* 173.8, 173, 155.5, 134.5, 130.3, 130, 129.3, 128.5, 124.0, 123.4, 121.5, 118.7, 117.5, 116.2, 111.3, 102.1, EI-MS: m/z calcd for C_16_H_11_N_3_OS [M]^+^ 293.0623, Found 293.0609.

#### Compound **4**: *3*-*(5*-*(indol*-*5*-*yl)*-*1,3,4*-*thiadiazol*-*2*-*yl)phenol*

Yield 81%, ^1^H-NMR (500 MHz, DMSO-*d*_*6*_): *δ* 9.60 (s,1H, NH), 8.34 (s, 1H, OH), 8.18 (s, 1H), 7.67 (d, 1H, *J* = 8.0 Hz), 7.48 (t, 2H, *J* = 7.5 Hz), 7.32 (d, 1H, *J* = 7.5 Hz), 7.24 (d, 1H, *J *= 7.5 Hz), 7.10 (d, 1H, *J* = 7.8), 6.83 (d, 1H, *J* = 7.5 Hz), 6.59 (s, 1H); ^13^C-NMR (125 MHz, DMSO-*d*_*6*_): δ 174.0, 174.0, 157.3, 151.4, 134.7, 130.4, 129.7, 128.0, 126.6, 123.3, 121.6, 115.7, 113.7, 112.6, 46.6, 30.4, EI-MS: m/z calcd for C_16_H_12_N_3_S [M]^+^ 293.0623, Found 293.0608.

#### Compound **5**: *2*-*(5*-*(1H*-*indol*-*5*-*yl)*-*1,3,4*-*thiadiazol*-*2*-*yl)benzene*-*1,4*-*diol*

Yield 80%, ^1^H-NMR (500 MHz, DMSO-*d*_*6*_): *δ* 11.92 (s, 1H, NH), 10.62 (s, 1H, OH), 8.42 (s, 1H, OH), 8.31 (s, 1H), 7.70 (d, 1H, *J* = 8.0 Hz), 7.49 (d, 2H, *J* = 8.0 Hz), 6.75 (s, 1H), 6.65 (s, 1H), 6.55 (d,1H, *J* = 8.0 Hz), 6.73 (d,1H, *J *= 8.0 Hz), 6.56 (s, 1H); ^13^C-NMR (125 MHz, DMSO-*d*_*6*_): *δ* 174.0, 174.0, 150.0, 147.5, 135.3, 129.5, 128.5, 125.0, 124.1, 118.8, 117.6, 117.1, 116.2, 114.1, 111.4, 102.2, EI-MS: m/z calcd for C_16_H_11_N_3_O_2_S [M]^+^ 309.0572, Found 309.0554.

#### Compound **6**: *3*-*(5*-*(1H*-*indol*-*5*-*yl)*-*1,3,4*-*thiadiazol*-*2*-*yl)benzene*-*1,2*-*diol*

Yield 88%, ^1^H-NMR (500 MHz, DMSO-*d*_*6*_*)*: *δ* 12.08 (s, 1H, NH), 9.14 (s, 1H, OH), 8.55 (s, 1H, OH), 8.20 (s, 1H), 7.70 (d, 1H, *J *= 8.0 Hz), 7.55 (d, 1H, *J* = 8.0 Hz), 6.90 (d, 1H, *J *= 7.8 Hz), 6.85 (d, 1H*, J* = 7.8), 6.76 (t, 1H, *J* = 7.0 Hz), 6.60 (d, 1H, *J* = 2.0 Hz); ^13^C-NMR (125 MHz, DMSO-*d*_*6*_): *δ* 174.0. 174.0, 145.4, 143.7, 135.3, 129.5, 128.5, 125.0, 124.1, 123.0, 121.3, 118.8, 117.1, 116.2, 111.4, 102.2, EI-MS: m/z calcd for C_16_H_11_N_3_O_2_S [M]^+^ 309.0572, Found 309.0550.

#### Compound **7**: *4*-*(5*-*(1H*-*indol*-*5*-*yl)*-*1,3,4*-*thiadiazol*-*2*-*yl)benzene*-*1,2*-*diol*

Yield 77%, ^1^H-NMR (500 MHz, DMSO-*d*_*6*_): *δ* 9.32 (s, 1H, NH), 9.21 (s, 1H, OH), 8.32 (s, 1H, OH), 8.6 (s, 1H),7.71 (d, 1H, *J* = 8.0 Hz), 7.42 (d, 2H, *J* = 8.0 Hz), 7.23 (s, 1H), 6.91 (d, 1H, *J* = 7.0 Hz), 6.74 (d, 1H, *J* = 8.0 Hz), 6.56 (d, 1H, *J* = 2.0 Hz); ^13^C-NMR (125 MHz, DMSO-*d*_*6*_): *δ* 174.0, 174.0, 147.1, 145.7, 135.4, 129.5, 128.5, 127.3, 124.1, 121.3, 118.8, 116.2, 116.0, 14.1, 111.4, 102.2, EI-MS: m/z calcd for C_16_H_11_N_3_O_2_S [M]^+^ 309.0572, Found 309.0559.

#### Compound **8**: *4*-*(5*-*(1H*-*indol*-*5*-*yl)*-*1,3,4*-*thiadiazol*-*2*-*yl)benzene*-*1,3*-*diol*

Yield 73%, ^1^H-NMR (500 MHz, DMSO-*d*_*6*_): *δ* 11.80 (s,1H, NH), 9.92 (s, 1H, OH), 8.53 (s, 1H, OH), 8.18 (s, 1H), 7.71 (d, 1H, *J *= 8.0 Hz), 7.47 (d, 2H, *J *= 8.0 Hz), 7.44 (t, 1H, *J *= 6.0 Hz), 7.25 (d, 1H, *J *= 8.0), 6.34 (d, 1H, *J *= 8.0 Hz), 6.31(d, 1H, *J *= 6.0 Hz); ^13^C-NMR (125 MHz, DMSO-*d*_*6*_): *δ* 174.0, 174.0, 159.7, 156.4, 135.3, 130.1, 129.5, 128.5, 124.1, 118.8, 116.2, 116.1, 111.4, 108.9, 105.4, 102.2, EI-MS: m/z calcd for C_16_H_11_N_3_O_2_S [M]^+^ 309.0572, Found 309.0558.

#### Compound **9**: *4*-*(5*-*(1H*-*indol*-*5*-*yl)*-*1,3,4*-*thiadiazol*-*2*-*yl)phenol*

Yield 79%, ^1^H-NMR (500 MHz, DMSO-*d*_*6*_): *δ* 11.59 (s, 1H, NH), 8.39 (s, 1H, OH), 8.16 (S, 1H), 7.65 (d, 1H, *J *= 8.0 Hz), 7.54 (d, 2H, *J *= 8.0 Hz), 7.45-7.42 (m, 2H), 6.80 (d, 2H, *J *= 8.0 Hz), 6.55 (s, 1H); ^13^C-NMR (125 MHz, DMSO-*d*_*6*_): *δ* 174.0, 174.0, 158.3, 135.3, 129.5, 128.7, 128.7, 128.5, 126.0, 124.1, 118.8, 116.2, 116.2, 116.2, 111.4, 102.2, EI-MS: m/z calcd for C_16_H_11_N_3_OS [M]^+^ 293.0623, Found 293.0627.

#### Compound **10**: *2*-*(1H*-*indol*-*5*-*yl)*-*5*-*(2*-*nitrophenyl)*-*1,3,4*-*thiadiazole*

Yield 81%, ^1^H-NMR (500 MHz, DMSO-*d6*): *δ* 12.10 (s, 1H, NH), 8.23 (s, 1H), 8.14 (d, 1H, *J* = 7.0 Hz), 8.05 (d, 1H, *J* = 8.0 Hz), 7.82 (t, 1H, *J* = 7.0 Hz), 7.70 (d, 1H, *J *= 8.0), 7.65 (t, 1H, *J* = 7.0 Hz), 7.51-7.48 (m, 2H); 6.72 (s, 1H); ^13^C NMR (125 MHz, DMSO-*d6*): *δ* 174.0, 174.0, 146.7, 135.3, 135.1, 131.4, 129.5, 129.4, 128.5, 128.2, 124.2, 124.1, 118.8, 116.2, 111.4, 102.2, EI-MS: m/z calcd for C_16_H_10_N_4_O_2_S [M]^+^ 322.0524, Found 322.0510.

#### Compound **11**: *2*-*(1H*-*indol*-*5*-*yl)*-*5*-*(3*-*nitrophenyl)*-*1,3,4*-*thiadiazole*

Yield 89%, ^1^H-NMR (500 MHz, DMSO-d6): *δ* 12.08 (s, 1H), 8.59 (d, 2H, *J* = 8.5 Hz), 8.25-8.22 (m, 2H), 8.14 (d, 1H, *J* = 7.0 Hz), 7.74 (t, 1H, *J* = 8.0 Hz), 7.70 (d, 1H, *J* = 8.0 Hz), 7.50-7.46 (m, 2H), 6.63 (s, 1H); ^13^C NMR (125 MHz, DMSO-*d6*): *δ* 174.0, 174.0, 148.2, 137.0, 135.3, 134.2, 130.0, 129.5, 128.5, 124.1, 123.7, 122.6, 118.8, 116.2, 111.4, 102.2, EI-MS: m/z calcd for C_16_H_10_N_4_O_2_S [M]^+^ 322.0524, Found 322.0507.

#### Compound **12**: *2*-*(1H*-*indol*-*5*-*yl)*-*5*-*(4*-*nitrophenyl)*-*1,3,4*-*thiadiazole*

Yield 91%, ^1^H-NMR (500 MHz, DMSO-*d6*): *δ* 12.03 (s, 1H, NH), 8.30 (d, 2H, *J *= 8.0 Hz), 8.22 (s, 1H), 8.15 (d, 1H, *J *= 8.0 Hz), 8.01 (d, 2H, *J *=8.0 Hz), 7.70 (d, 1H, *J *= 8.0 Hz), 7.49-7.46 (m, 2H) 6.62 (s, 1H); ^13^C NMR (125 MHz, DMSO-d6): δ 174.7, 174, 148.1, 139.2, 135.2, 129.5, 128.6, 128.7, 128.2, 124.8, 124.5, 124, 119.3, 116, 111.2, 102, EI-MS: m/z calcd for C_16_H_10_N_4_O_2_S [M]^+^ 322.0524, Found 322.0509.

#### Compound **13**: *2*-*(5*-*(1H*-*indol*-*5*-*yl)*-*1,3,4*-*thiadiazol*-*2*-*yl)*-*4*-*methoxyphenol*

Yield 90%, ^1^H-NMR (500 MHz, DMSO-*d6*): *δ* 12.08 (s, 1H, NH), 11.41 (s, 1H, OH), 8.23 (s, 1H), 7.70 (d, 1H, *J *= 8.0 Hz), 7.49-7.46 (m, 2H), 7.09 (d, 1H, *J *= 8.0 Hz), 6.90 (d, 2H, *J *= 2.0 Hz), 6.84 (d, 1H, *J *= 7.5 Hz), 3.78 (s, 3H); ^13^C NMR (125 MHz, DMSO-*d6*): *δ* 174.7, 174.0, 153.5, 147.1, 135.8, 129.9, 128.6, 124.9, 124.4, 119.3, 117.2, 116.2, 115.4, 112.5, 111.3, 102.2, 55.4, EI-MS: m/z calcd for C_17_H_13_N_3_O_2_S [M]^+^ 323.0728, Found 323.0711.

#### Compound **14**: *2*-*(3*-*bromo*-*4*-*fluorophenyl)*-*5*-*(1H*-*indol*-*5*-*yl)*-*1,3,4*-*thiadiazole*

Yield 88%, ^1^H-NMR (500 MHz, DMSO-*d6*): *δ* 11.94 (s, 1H), 8.20 (s, 1H), 8.02 (d, 1H, *J *= 7.0 Hz), 7.75 (s, 1H), 7.67 (d, 2H, *J *= 7.0 Hz), 7.48-7.45 (m, 3H), 6.65 (s, 1H); ^13^C NMR (125 MHz, DMSO-*d6*): *δ* 174.4, 174.3, 165.2, 135.3, 134.4, 131, 129.4, 128.5, 128.8, 124.7, 119.3, 118, 116.3, 111.3, 110.4, 102.2, EI-MS: m/z calcd for C_16_H_9_BrFN_3_S [M]^+^ 372.9685, Found 372.9661.

#### Compound **15**: *2*-*(3*-*chlorophenyl)*-*5*-*(1H*-*indol*-*5*-*yl)*-*1,3,4*-*thiadiazole*

Yield 90%, ^1^H-NMR (500 MHz, DMSO-*d6*): *δ* 9.72 (s, 1H, NH), 8.20 (s, 1H), 7.78 (d, 1H, *J *= 7.0 Hz), 7.48-7.45 (m, 2H), 7.35-7.31 (m, 2H), 7.12 (d, 1H, *J *= 7.0 Hz), 6.82 (d, 1H, *J *= 8.0 Hz), 6.62 (s, 1H); ^13^C NMR (125 MHz, DMSO-*d6*): *δ* 174.7, 174.4, 135.3, 135.1, 134.5, 129.5, 129.4, 129.3, 128.5, 128.3, 127.2, 124.1, 119.3, 116.2, 111.7, 102.9, EI-MS: m/z calcd for C_16_H_10_ClN_3_S [M]^+^ 311.0284, Found 311.0280.

#### Compound **16**: *2*-*(1H*-*indol*-*5*-*yl)*-*5*-*(pyridin*-*3*-*yl)*-*1,3,4*-*thiadiazole*

Yield 81%, ^1^H-NMR (500 MHz, DMSO-*d6*): *δ* 11.90 (s, 1H), 8.62 (s, 1H),8.58 (d, 1H, *J *= 5.0 Hz), 8.24 (s, 1H), 8.12 (d, 1H, *J *=6.5 Hz), 7.70 (d, 1H, *J *= 8.0 Hz), 7.46-7.42 (m, 3H), 6.58 (s, 1H); ^13^C NMR (125 MHz, DMSO-*d6*): *δ* 174.4, 174, 149.3, 148.1, 135.2, 134.3, 133.2, 129.5, 128.4, 124.4, 124.3, 119.2, 116.2, 111.4, 102.6, EI-MS: m/z calcd for C_15_H_10_N_4_S [M]^+^ 278.0626, Found 278.0612.

#### Compound **17**: *2*-*(1H*-*indol*-*5*-*yl)*-*5*-*(pyridin*-*4*-*yl)*-*1,3,4*-*thiadiazole*

Yield 80%, ^1^H-NMR (500 MHz, DMSO-*d6*): *δ* 12.01 (s, 1H), 8.62 (d, 2H, *J *= 6.5 Hz), 8.48 (s, 1H), 7.70 (d, 1H, *J *= 8.0 Hz), 7.68 (d, 2H*, J *= 8.0 Hz), 7.57 (d, 1H, *J *= 4.5 Hz), 7.50 (d, 1H, *J *= 8.0 Hz), 6.66 (s, 1H); ^13^C NMR (125 MHz, DMSO-*d6*): *δ* 174.5, 174, 150.1, 149.5, 143.5, 135.3, 129.4, 128.4, 124.1, 121.4, 121.2, 119.2, 116.2, 111.4, 102.7, EI-MS: m/z calcd for C_15_H_10_N_4_S [M] + 278.0626, Found 278.0614.

#### Compound **18**: *2*-*(2*-*fluorophenyl)*-*5*-*(1H*-*indol*-*5*-*yl)*-*1,3,4*-*thiadiazole*

Yield 89%, ^1^H-NMR (500 MHz, DMSO-*d6*): *δ* 11.88 (s, 1H, NH), 8.22 (s, 1H), 8.08 (s, 1H), 7.70 (d, 1H, *J *= 8.0 Hz), 7.50 (d, 1H, *J *= 8.0 Hz), 7.42 (t, 1H, *J *= 6.5 Hz), 7.38 (t, 1H, *J *= 6.5 Hz), 7.29 (d, 2H, *J *= 8.0 Hz), 6.68 (s, 1H); ^13^C NMR (125 MHz, DMSO-*d6*): *δ* 174.4, 174.2, 158.1, 135.2, 130.1, 129.5, 129.4, 128.6, 124.7, 124.4, 123.4, 119.1, 116.5, 114.8, 111.4, 102.2, EI-MS: m/z calcd for C_16_H_10_FN_3_S [M] + 295.0559, Found 295.0574.

#### Compound **19**: *2*-*(3*-*fluorophenyl)*-*5*-*(1H*-*indol*-*5*-*yl)*-*1,3,4*-*thiadiazole*

Yield 83%, ^1^H-NMR (500 MHz, DMSO-*d6*): *δ*11.84 (s, 1H, NH), 8.44 (s, 1H), 8.20 (s, 1H), 7.68 (d, 1H, *J *= 8.0 Hz), 7.47-7.42(m, 4H), 7.26 (t, 1H, *J *= 8.0 Hz), 6.66 (s, 1H); *δ* ^13^C NMR (125 MHz, DMSO-*d6*): *δ* 174.6, 174.2, 162.4, 135.8, 135.6, 129.5, 128.4, 127.3, 126.2, 124.1, 119.3, 116.2, 116.2, 115.3, 111.4, 102.2, EI-MS: m/z calcd for C_16_H_10_FN_3_S [M] + 295.0579, Found 295.057.

#### Compound **20**: *2*-*(4*-*fluorophenyl)*-*5*-*(1H*-*indol*-*5*-*yl)*-*1,3,4*-*thiadiazole*

Yield 88%, ^1^H-NMR (500 MHz, DMSO-*d6*): *δ* 11.82 (s, 1H, NH), 8.15 (s, 1H), 7.64 (d, 2H, *J *= 8.0 Hz),7.49 (t, 1H, *J *= 8.0 Hz),7.47 (t, 2H, *J *=5.5 Hz), 7.31 (t, 2H, *J *= 8.0 Hz), 6.58 (s, 1H); ^13^C NMR (125 MHz, DMSO-*d6*): *δ* 174.4, 174.2, 162.7, 135.2, 129.4, 129.4, 129.2, 129, 128.5, 124.1, 119.6, 117.2, 116.7, 116.2, 111.4, 102.2, EI-MS: m/z calcd for C_16_H_10_FN_3_S [M] + 295.0579, Found 295.0574.

#### Compound **21**: *2*-*(2*-*chlorophenyl)*-*5*-*(1H*-*indol*-*5*-*yl)*-*1,3,4*-*thiadiazole*

Yield 83%, ^1^H-NMR (500 MHz, DMSO-*d6*): *δ* 12.03 (s, 1H, NH), 8.23 (s, 1H), 8.05 (s, 1H), 7.70 (d, 1H, *J *= 8.0 Hz), 7.50 (t, 1H, *J *= 8.0 Hz), 7.47 (d, 1H, *J *=8.0 Hz), 7.42 (t, 1H, *J *= 5.0 Hz), 7.39 (d, 2H*, J *= 6.5 Hz), 6.56 (s, 1H); ^13^C NMR (125 MHz, DMSO-*d6*): *δ* 174.7, 174.3, 137.1, 135.3, 132.0, 130.3, 129.5, 129.5, 129.2, 128.5, 127.1, 124.2, 119.1, 116.2, 111.4, 102.2, EI-MS: m/z calcd for C_16_H_10_ClN_3_S [M]^+^ 311.0284, Found 311.0267.

#### Compound **22**: *2*-*(4*-*chlorophenyl)*-*5*-*(1H*-*indol*-*5*-*yl)*-*1,3,4*-*thiadiazole*

Yield 81%, ^1^H-NMR (500 MHz, DMSO-*d6*): *δ* 11.80 (s, 1H, NH), 8.20 (s, 1H), 7.74 (d, 2H, *J *= 7.8 Hz), 7.68 (d, 1H, *J *= 8.0 Hz), 7.52 (d, 2H, *J *=8.0 Hz), 7.48 (d, 1H, *J *= 8.0 Hz), 7.43 (t, 1H, *J *= 6.0 Hz), 6.58 (s, 1H); ^13^C NMR (125 MHz, DMSO-*d6*): *δ* 174.6, 174.3, 135.3, 134.1, 131.4, 129.5, 129.4, 129.2, 129.0, 128.7, 128.4, 124.1, 119.3, 116.2, 111.4, 102.2, EI-MS: m/z calcd for C_16_H_10_ClN_3_S [M]^+^ 311.0284, Found 311.0260.

## Conclusion

In conclusion we have synthesized **22** derivatives of indole based thiadiazole hybrid analogs due to greater biological importance of indole and thiadiazole and evaluated for β-glucuronidase inhibition. All compounds showed outstanding β-glucuronidase activity ranging between 0.5 ± 0.08 to 38.9 ± 0.8 µM when compared with standard d-saccharic acid 1,4 lactone. Structure activity relationship has been established for all compounds which reveal that the number, nature and position of substituents on phenyl ring play of thiadiazole play a vital role in this inhibition. Molecular docking study have been performed which revealed that these compounds established stronger hydrogen bonding networks with active site residues.

## Additional file

**Additional file 1.** Supporting data having proton NMR of all compounds.
