# Supplementary material for: Indole bearing thiadiazole analogs: synthesis, β-glucuronidase inhibition and molecular docking study
Source: BMC Chem. 2019 Feb 4;13(1):14. doi: 10.1186/s13065-019-0522-x (PMC6661955; doi:10.1186/s13065-019-0522-x)
Supplement: Supplementary file 1 — Additional file 1. Supporting data having proton NMR of all compounds. [file 13065_2019_522_MOESM1_ESM.pdf]

Compound # 1

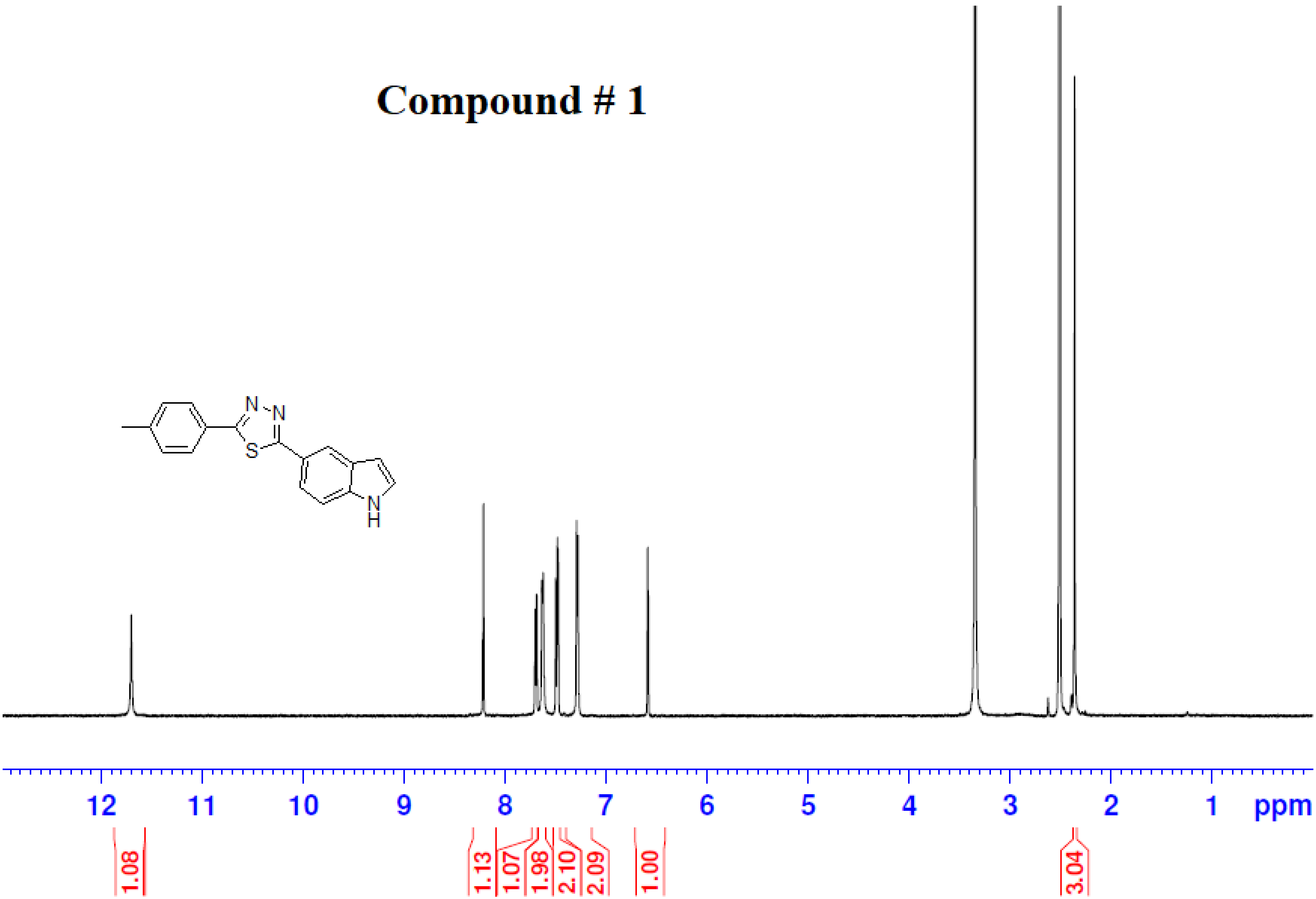

Compound # 2

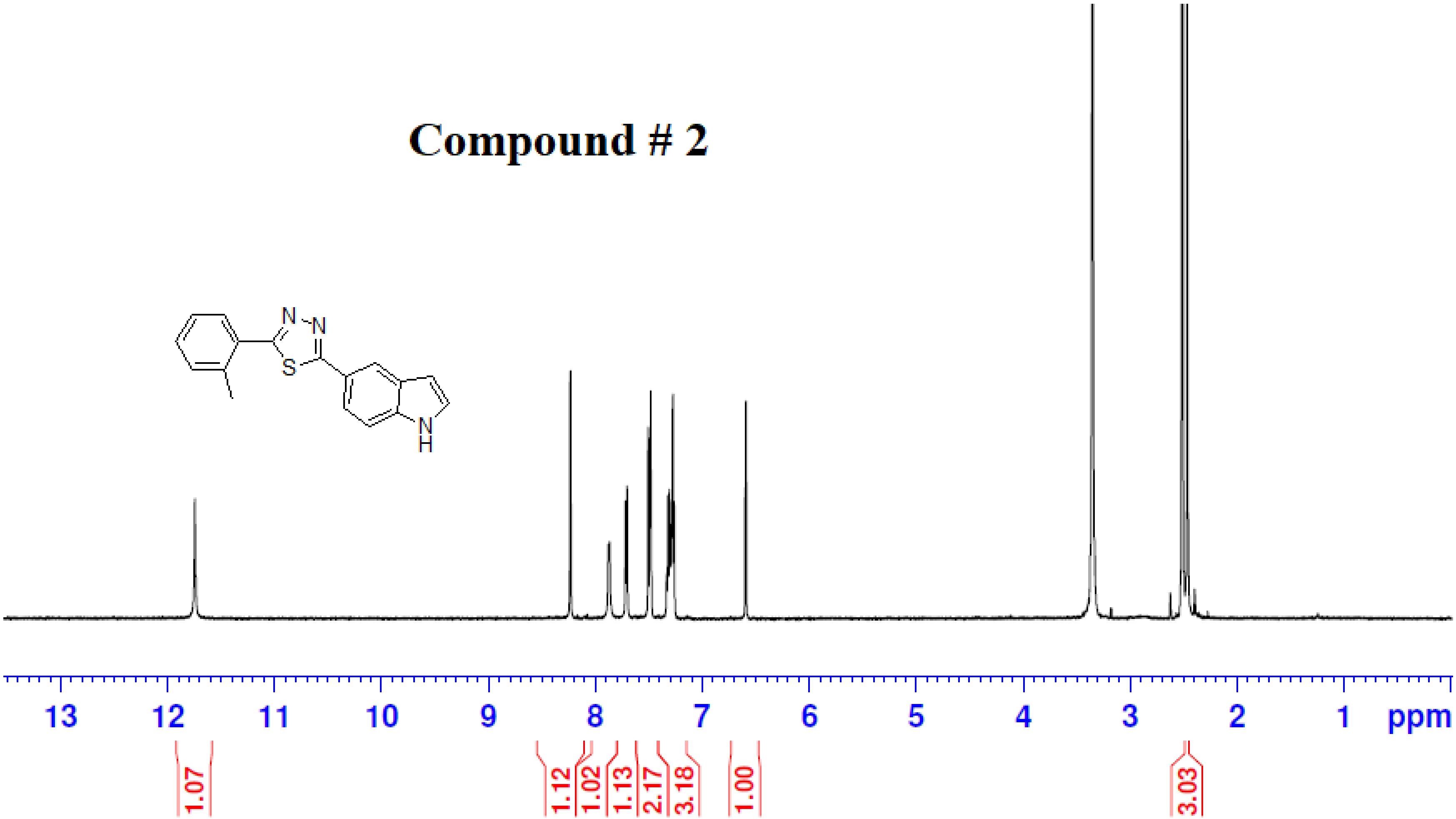

Compound # 3

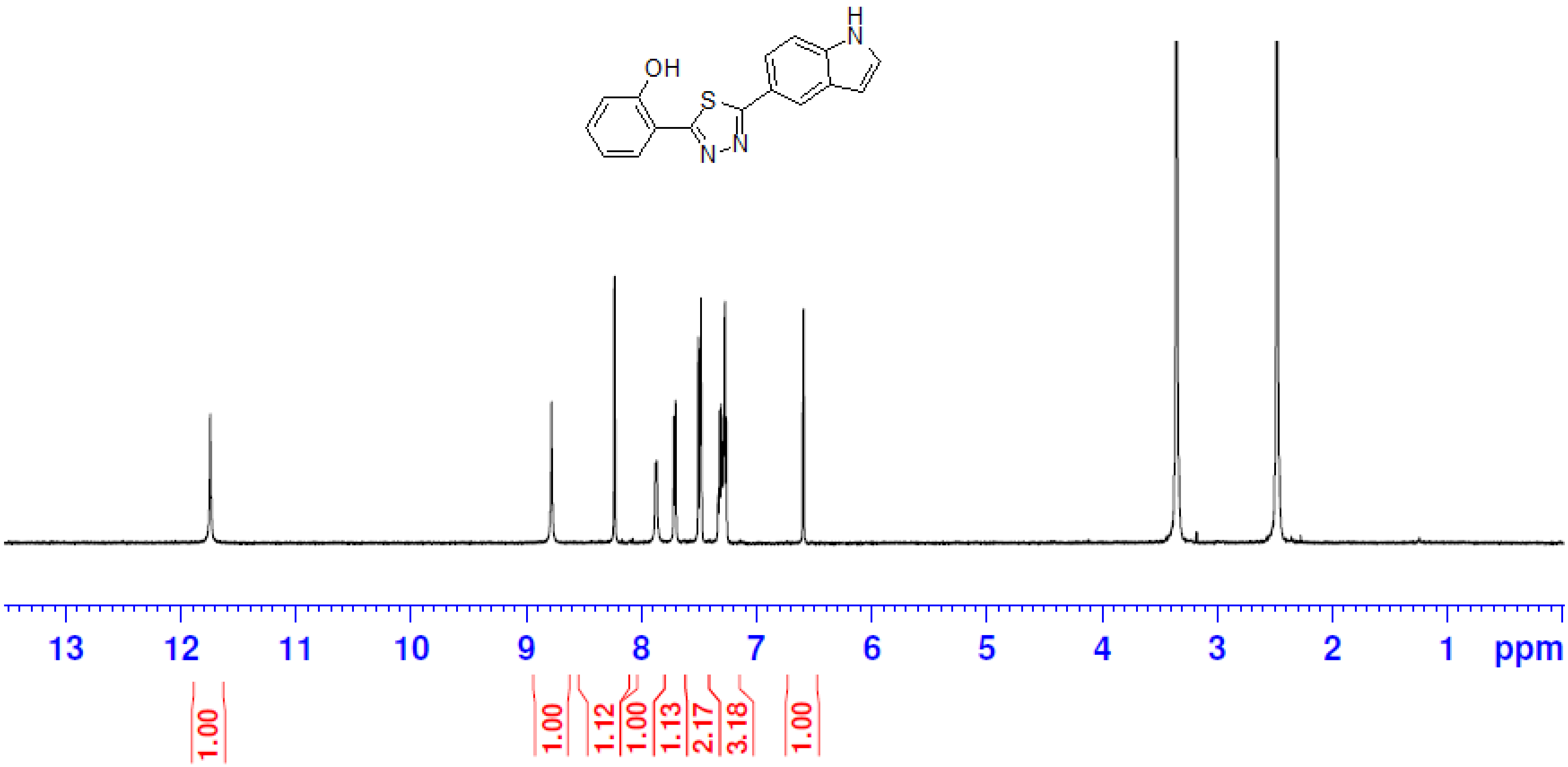

OH
S
N
N
H
N
13
12
11
10
9
8
7
6
5
4
3
2
1
ppm
1.00
1.00
1.12
1.00
1.13
2.17
3.18
1.00

Compound # 4

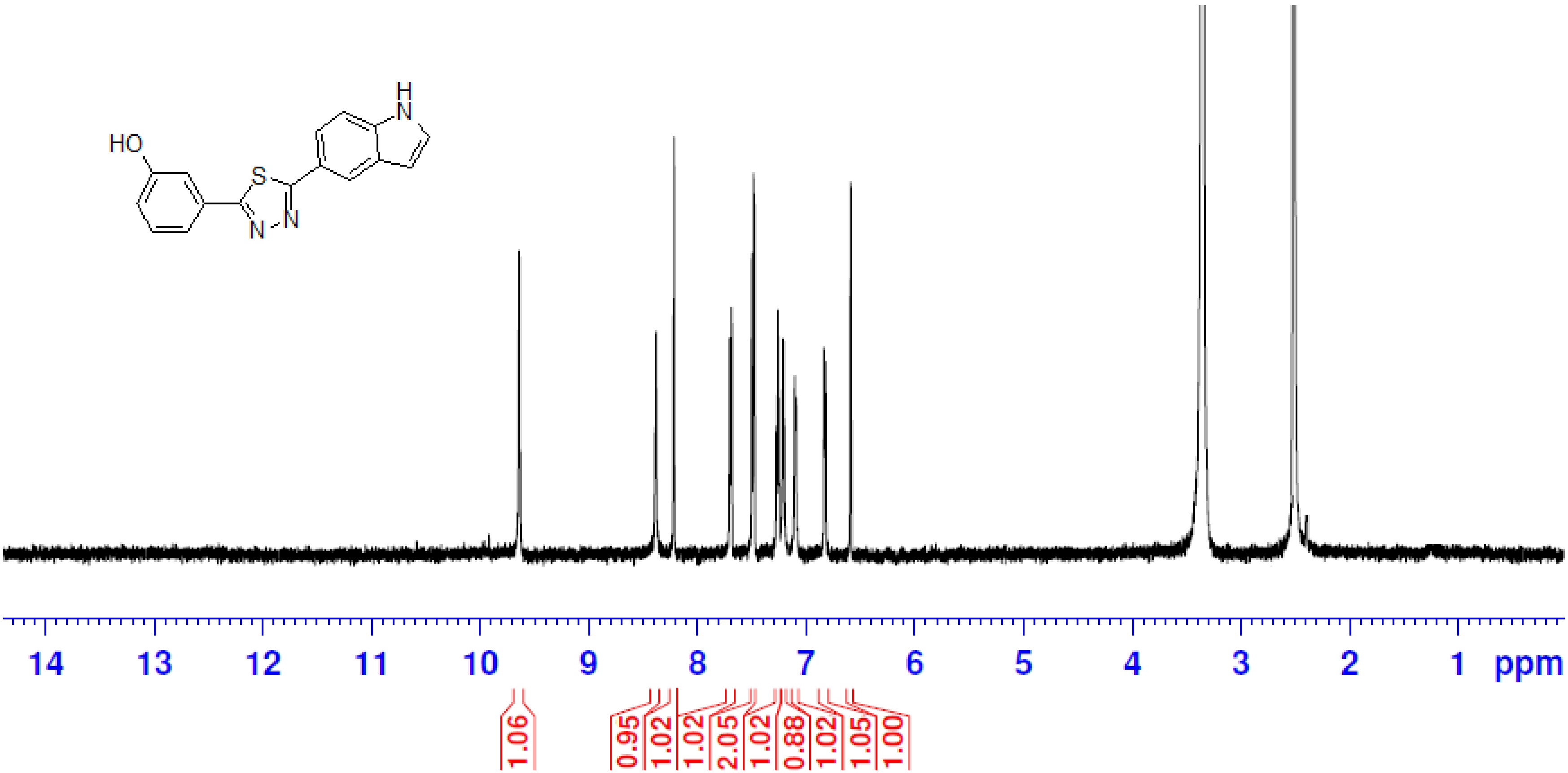

HO
S
N
N
H
N
1.06
0.95
1.02
1.02
2.05
1.02
0.88
1.02
1.05
1.00
14
13
12
11
10
9
8
7
6
5
4
3
2
1
ppm

Compound # 5

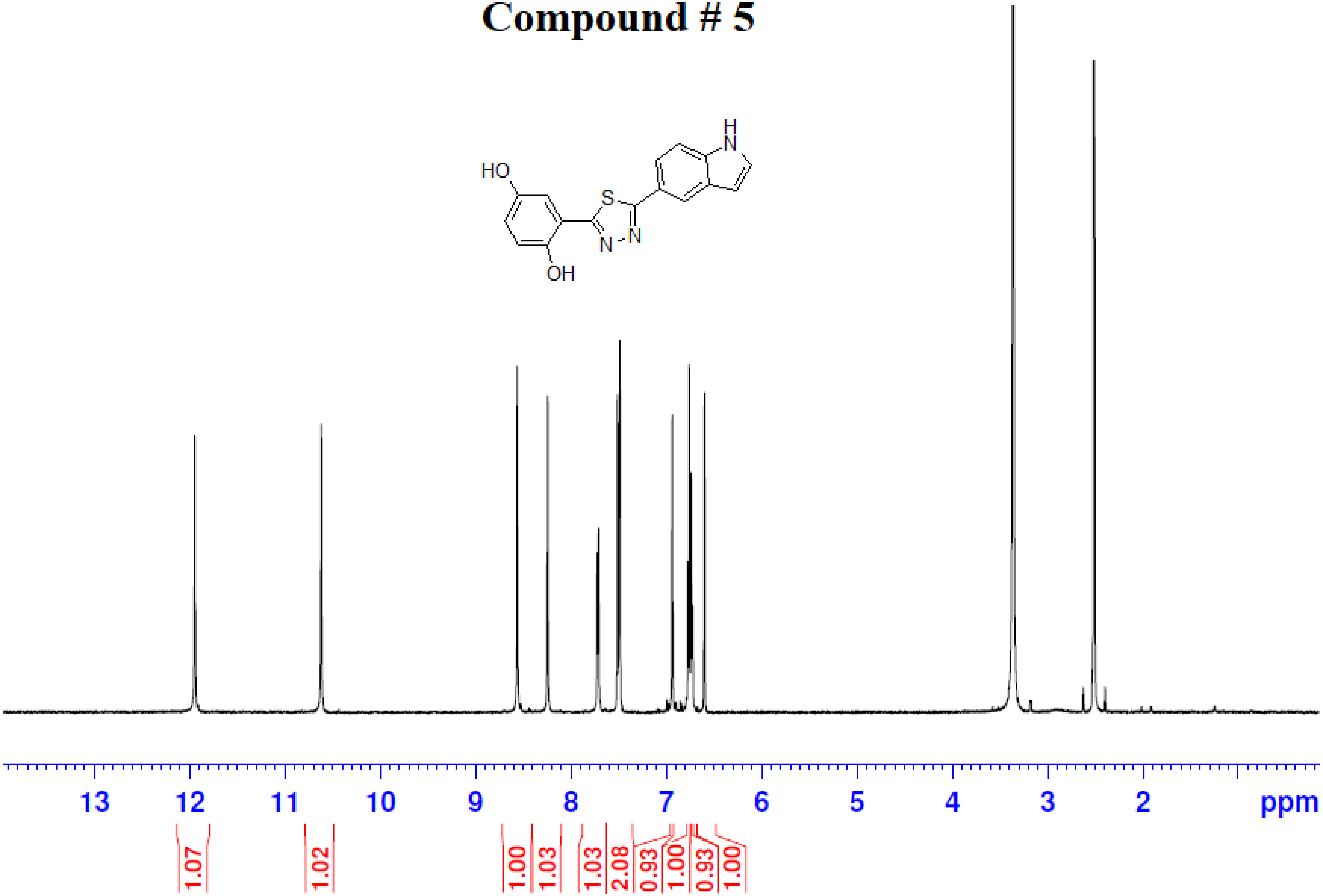
HO
OH
S
N
N
H
N
13
12
11
10
9
8
7
6
5
4
3
2
ppm
1.07
1.02
1.00
1.03
1.03
2.08
0.93
1.00
0.93
1.00

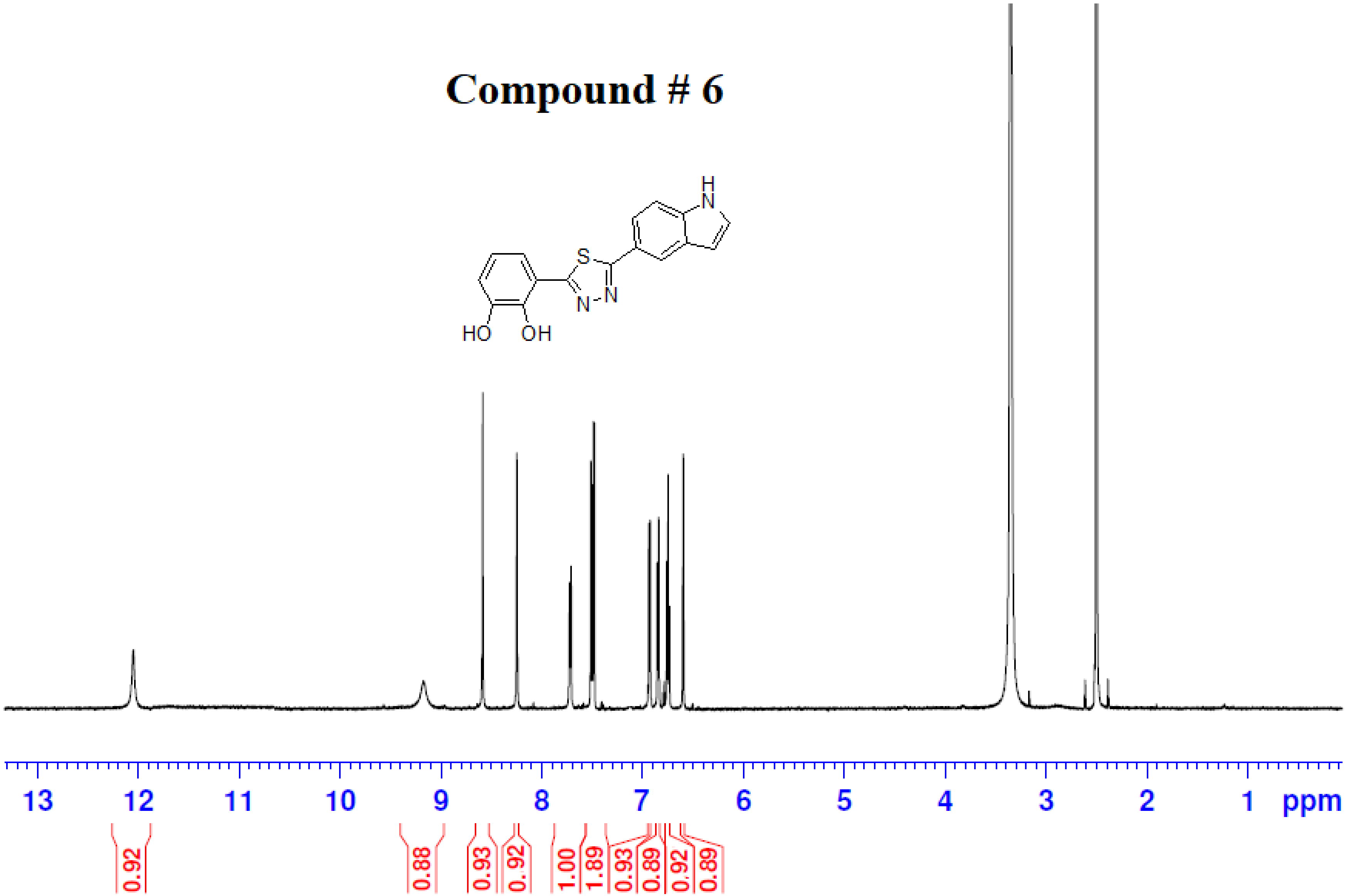
Compound # 6
HO
OH
S
N
N
H
N
13
12
11
10
9
8
7
6
5
4
3
2
1
ppm
0.92
0.88
0.93
0.92
1.00
1.89
0.93
0.89
0.92
0.89

Compound # 7

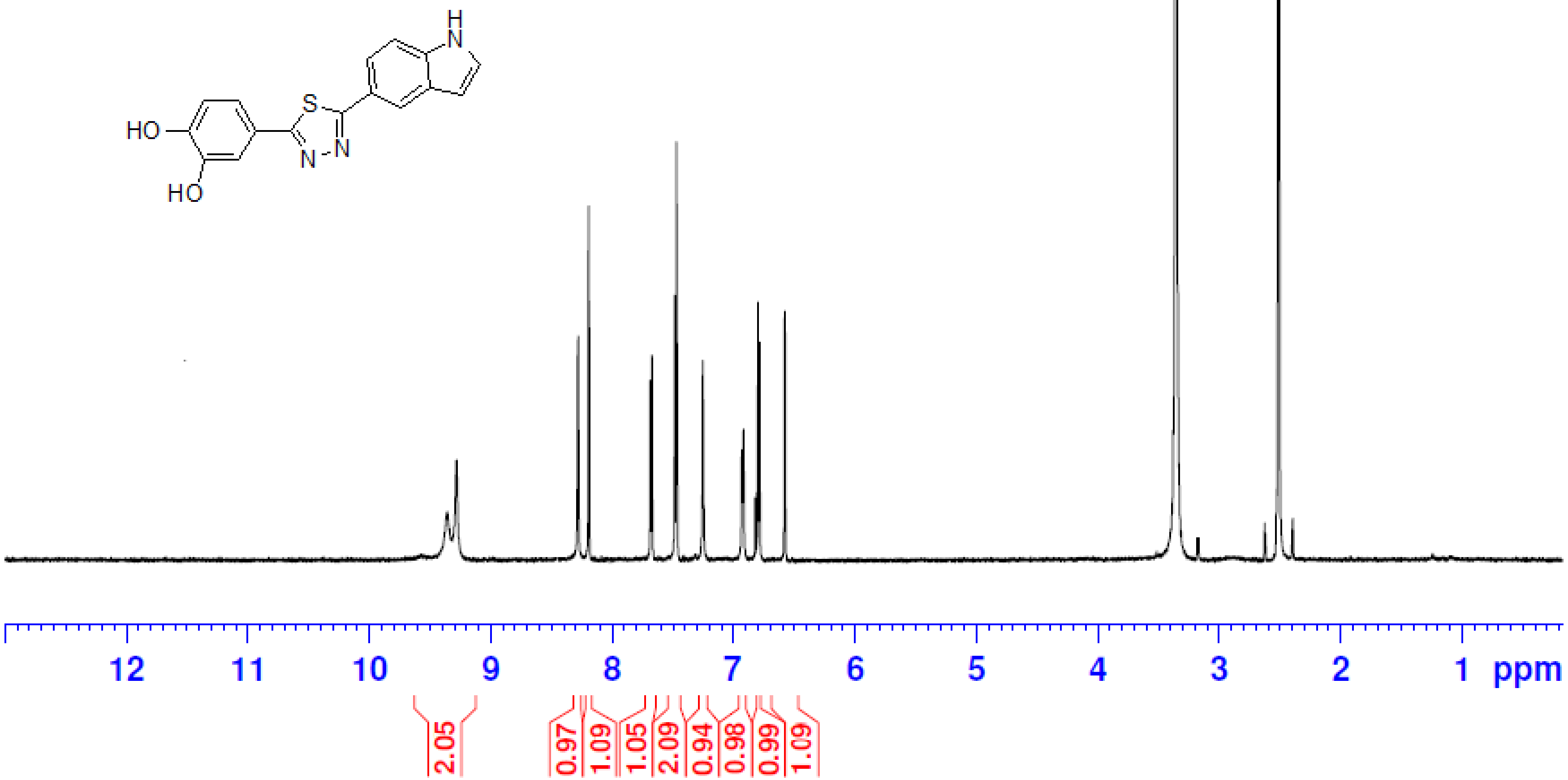

HO
HO
S
N
N
H
N
12
11
10
9
8
7
6
5
4
3
2
1
ppm
2.05
0.97
1.09
1.05
2.09
0.94
0.98
0.99
1.09

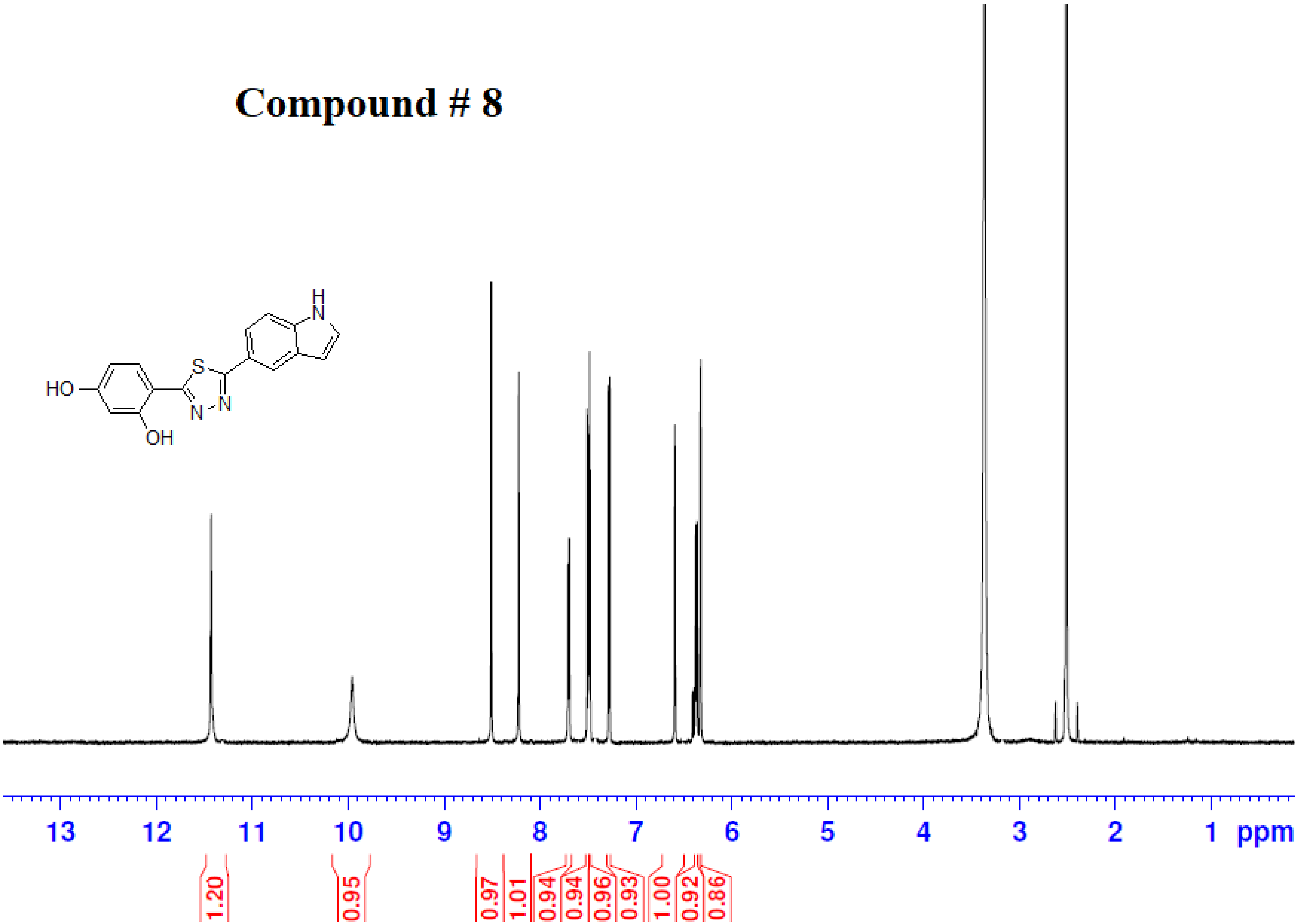

Compound # 8
HO
OH
S
N
N
H
N
13
12
11
10
9
8
7
6
5
4
3
2
1
ppm
1.20
0.95
0.97
1.01
0.94
0.94
0.96
0.93
1.00
0.92
0.86

Compound # 9

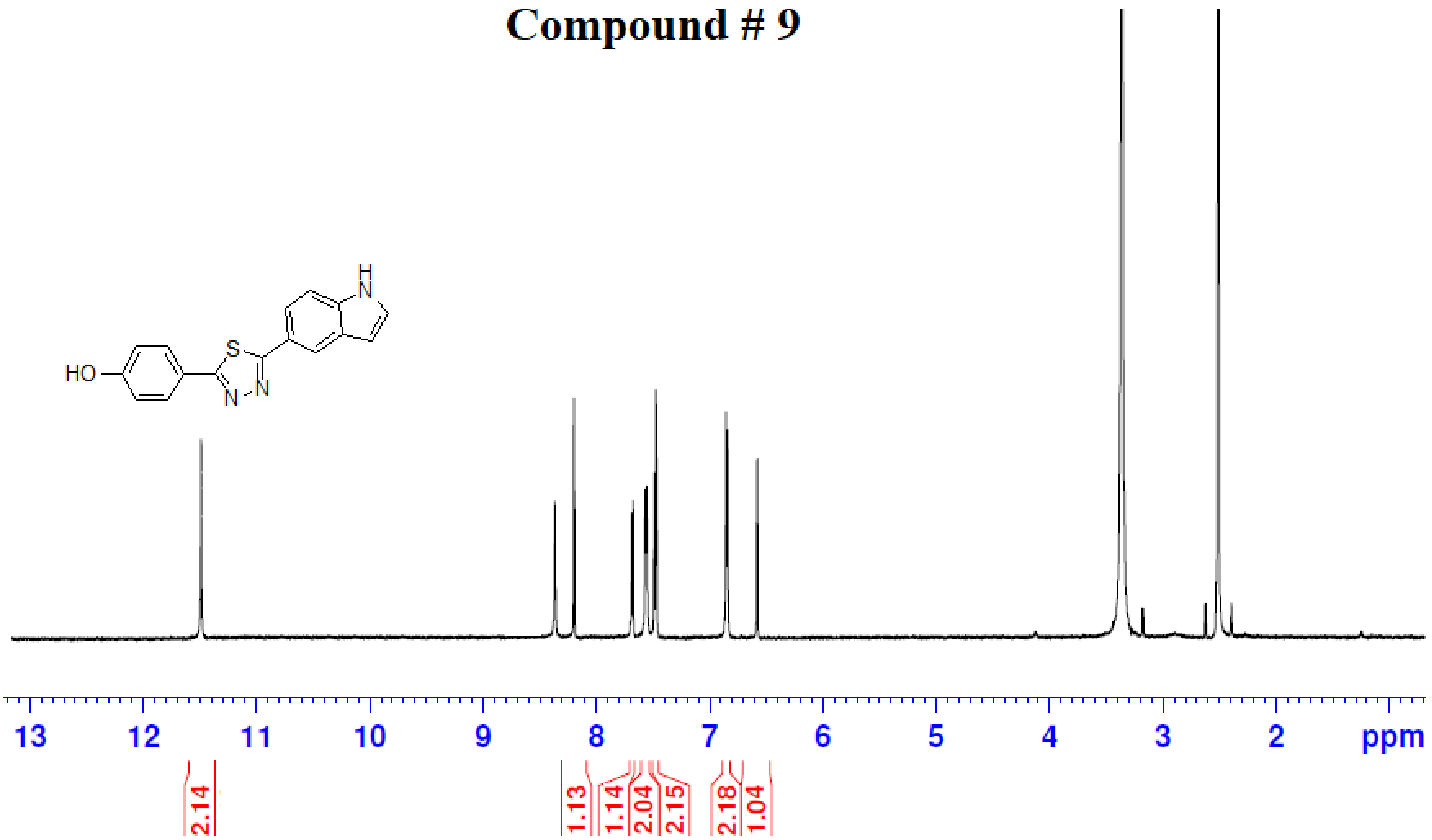

Compound # 10

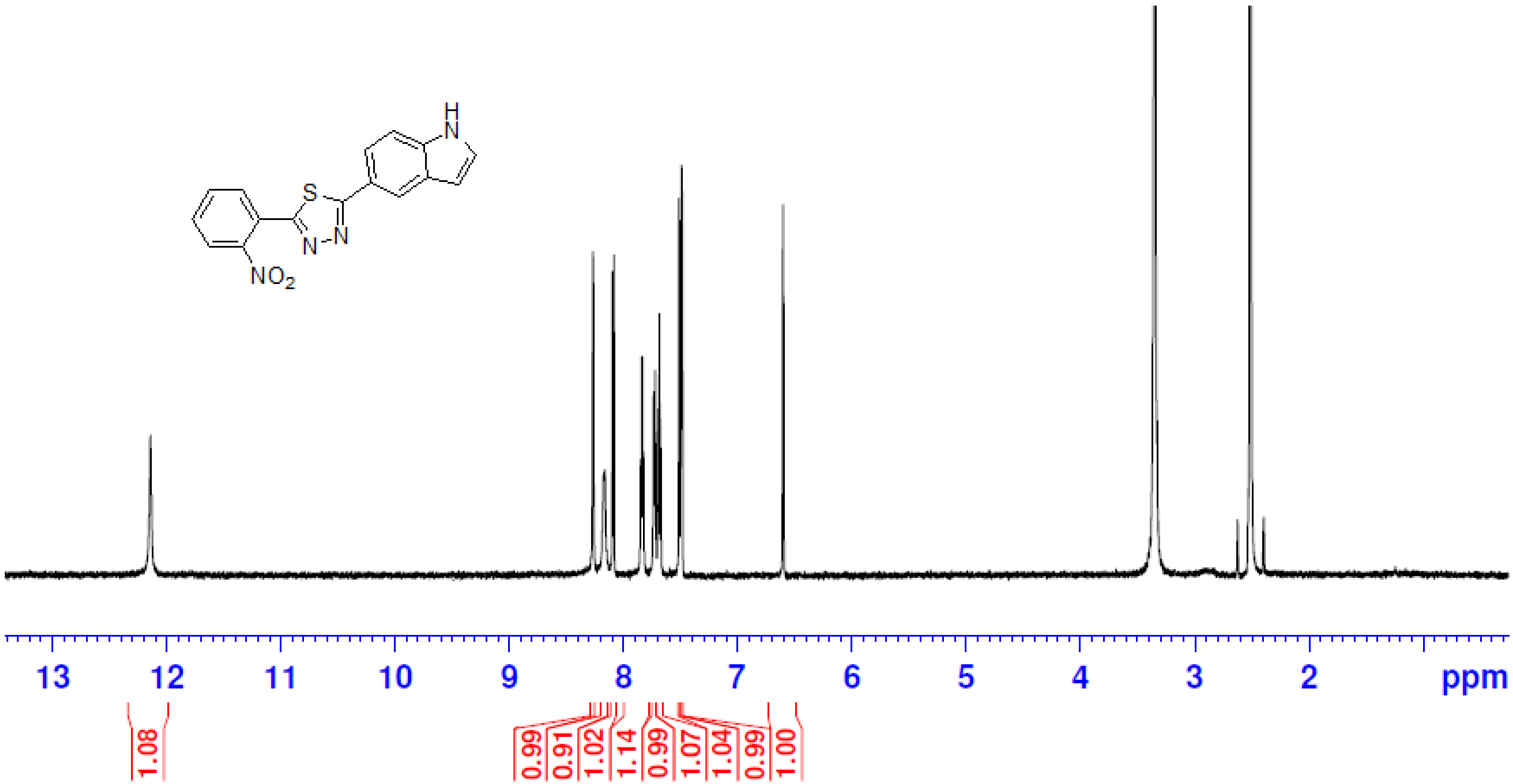
H
N
S
N
N
NO2
13
12
11
10
9
8
7
6
5
4
3
2
ppm
1.08
0.99
0.91
1.02
1.14
0.99
1.07
1.04
0.99
1.00

Compound # 11

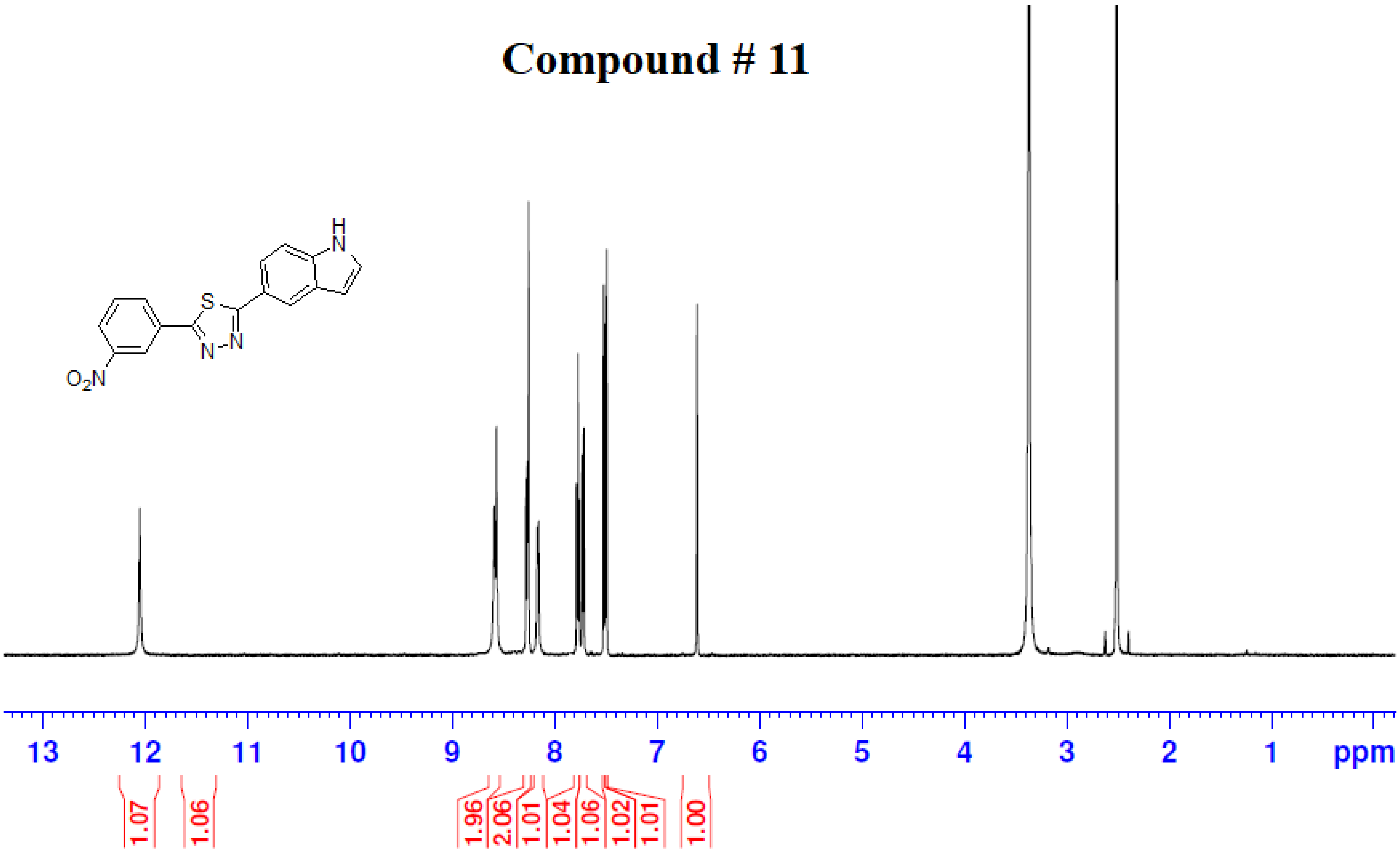

H
N
S
N-N
O2N
13 12 11 10 9 8 7 6 5 4 3 2 1 ppm
1.07
1.06
1.96
2.06
1.01
1.04
1.06
1.02
1.01
1.00

Compound # 12

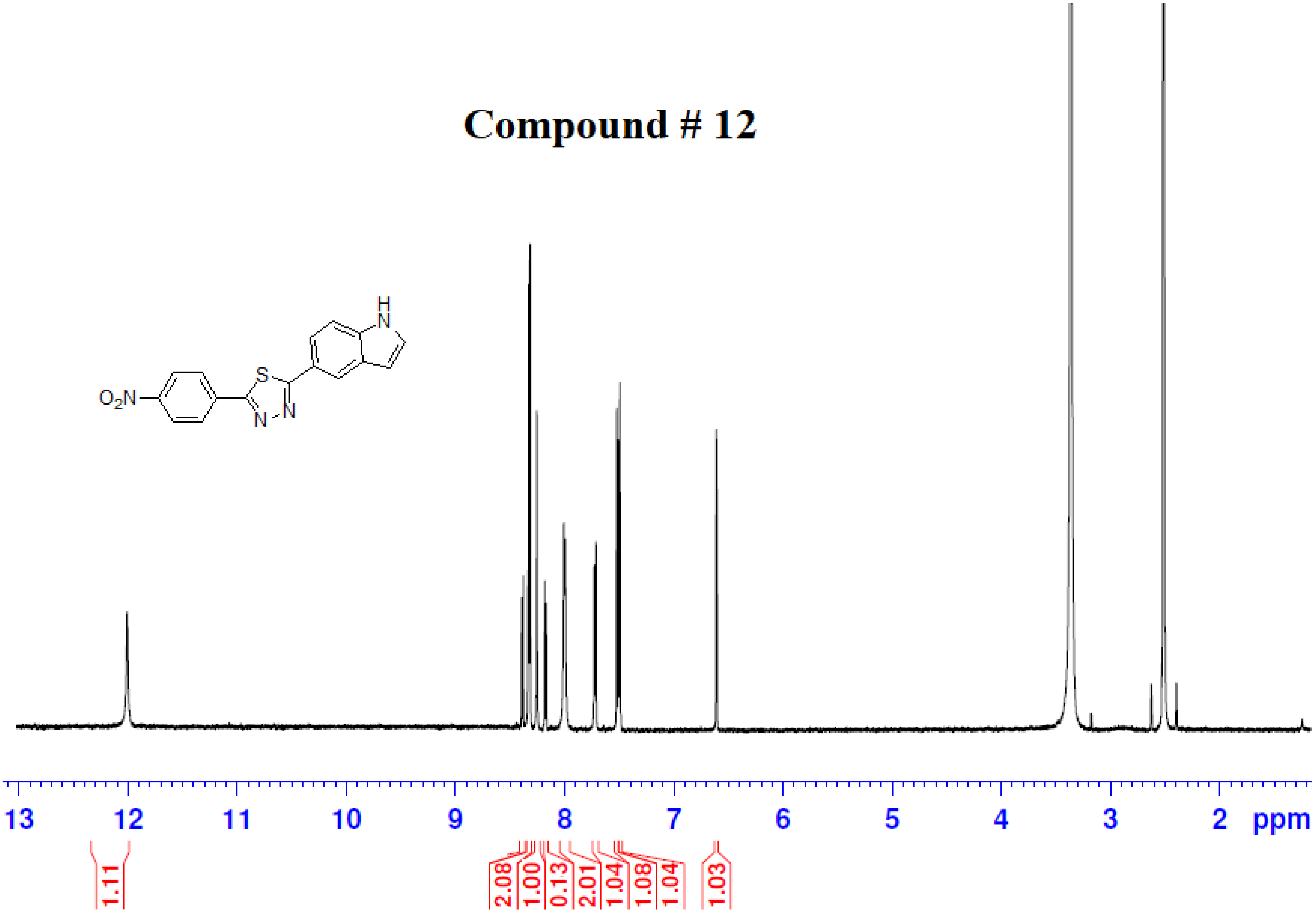

O2N
S
N
N
H
N
13
12
11
10
9
8
7
6
5
4
3
2
ppm
1.11
2.08
1.00
0.13
2.01
1.04
1.08
1.04
1.03

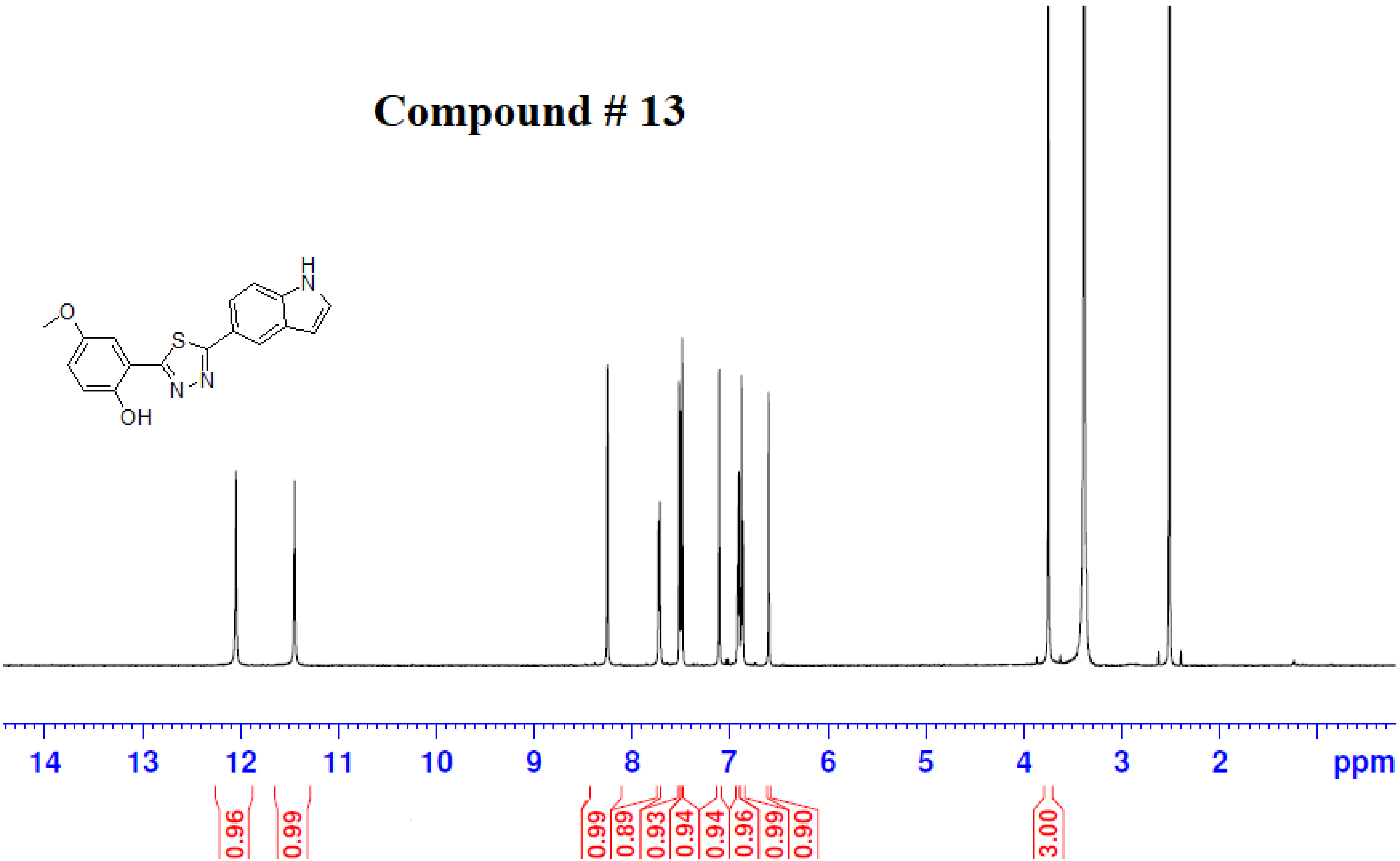
Compound # 13
O
S
N
N
H
N
OH
14
13
12
11
10
9
8
7
6
5
4
3
2
ppm
0.96
0.99
0.99
0.89
0.93
0.94
0.94
0.96
0.99
0.90
3.00

Compound # 14

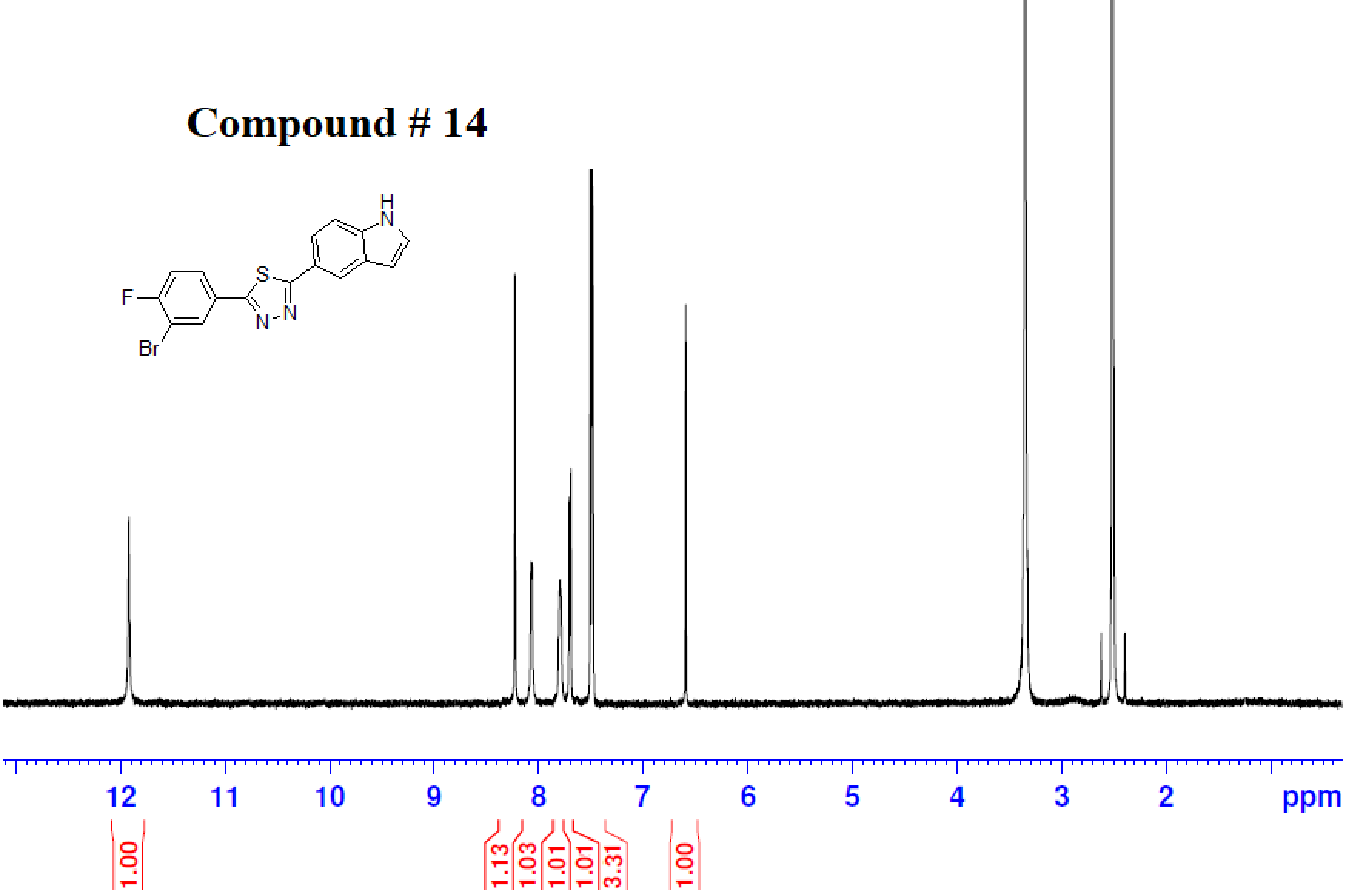

Compound # 15

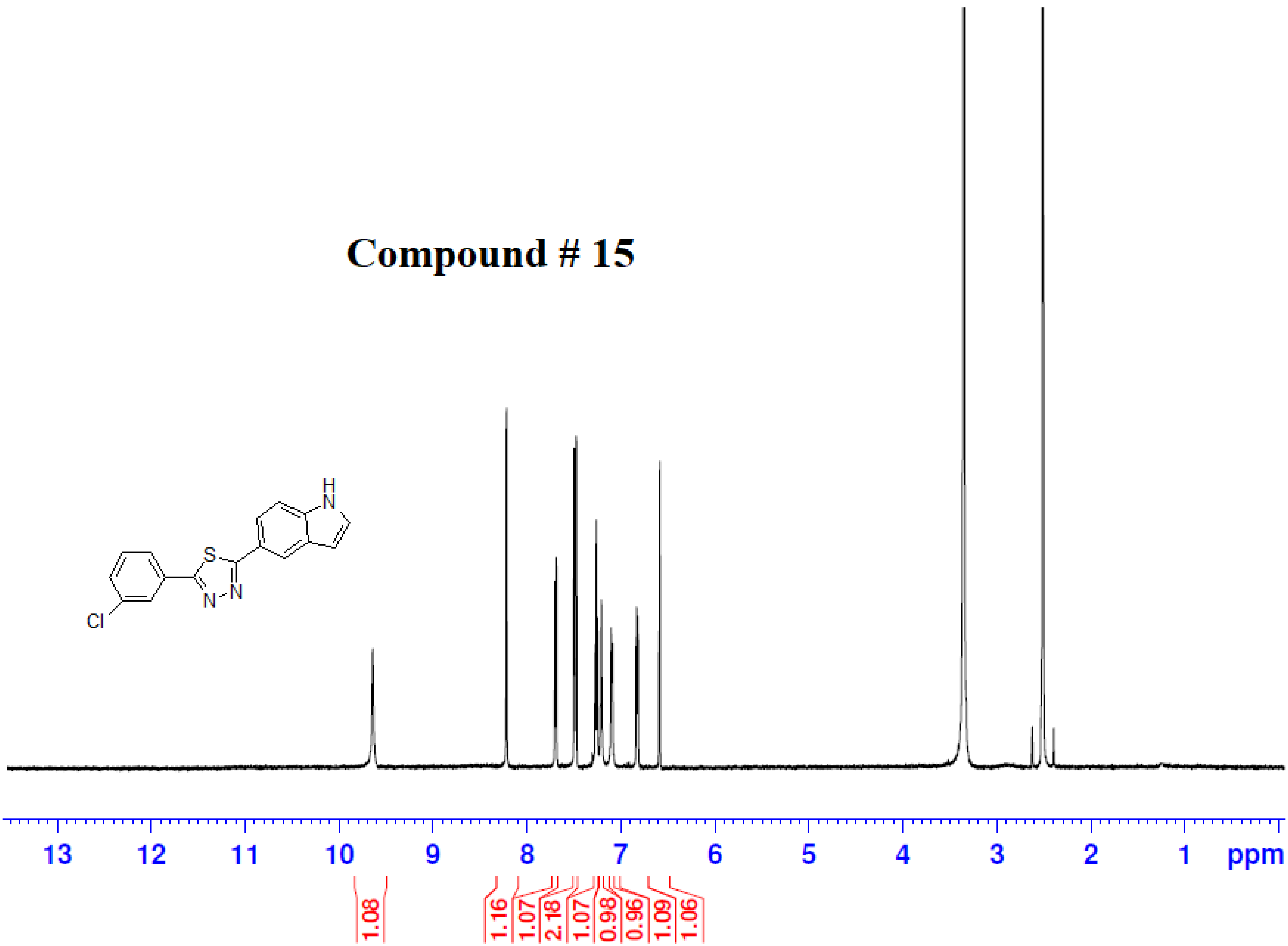

H
N
S
N
N
Cl
13
12
11
10
9
8
7
6
5
4
3
2
1
ppm
1.08
1.16
1.07
2.18
1.07
0.98
0.96
1.09
1.06

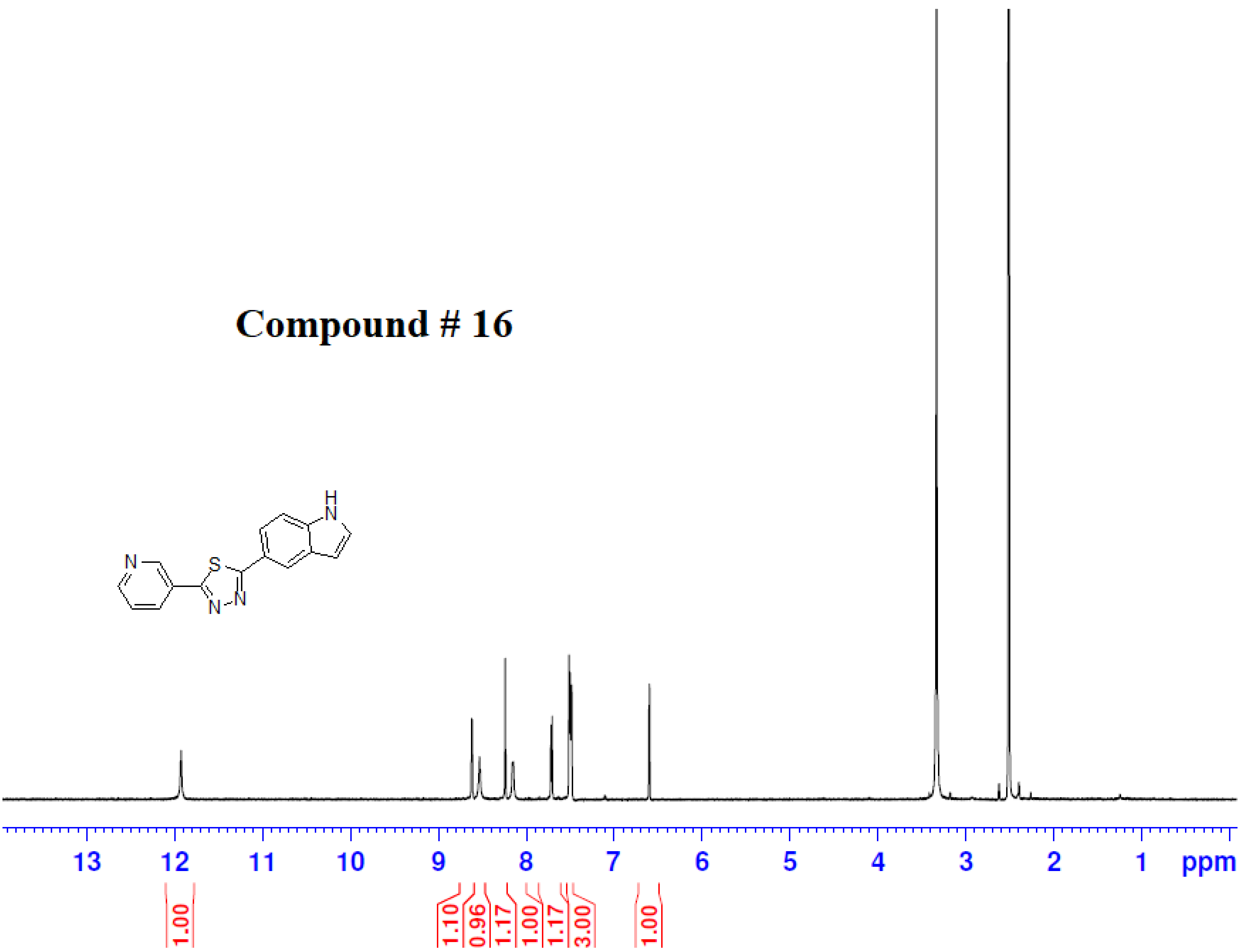

Compound # 16
N
S
N
N
H
N
13 12 11 10 9 8 7 6 5 4 3 2 1 ppm
1.00
1.10
0.96
1.17
1.00
1.17
3.00
1.00

Compound # 17

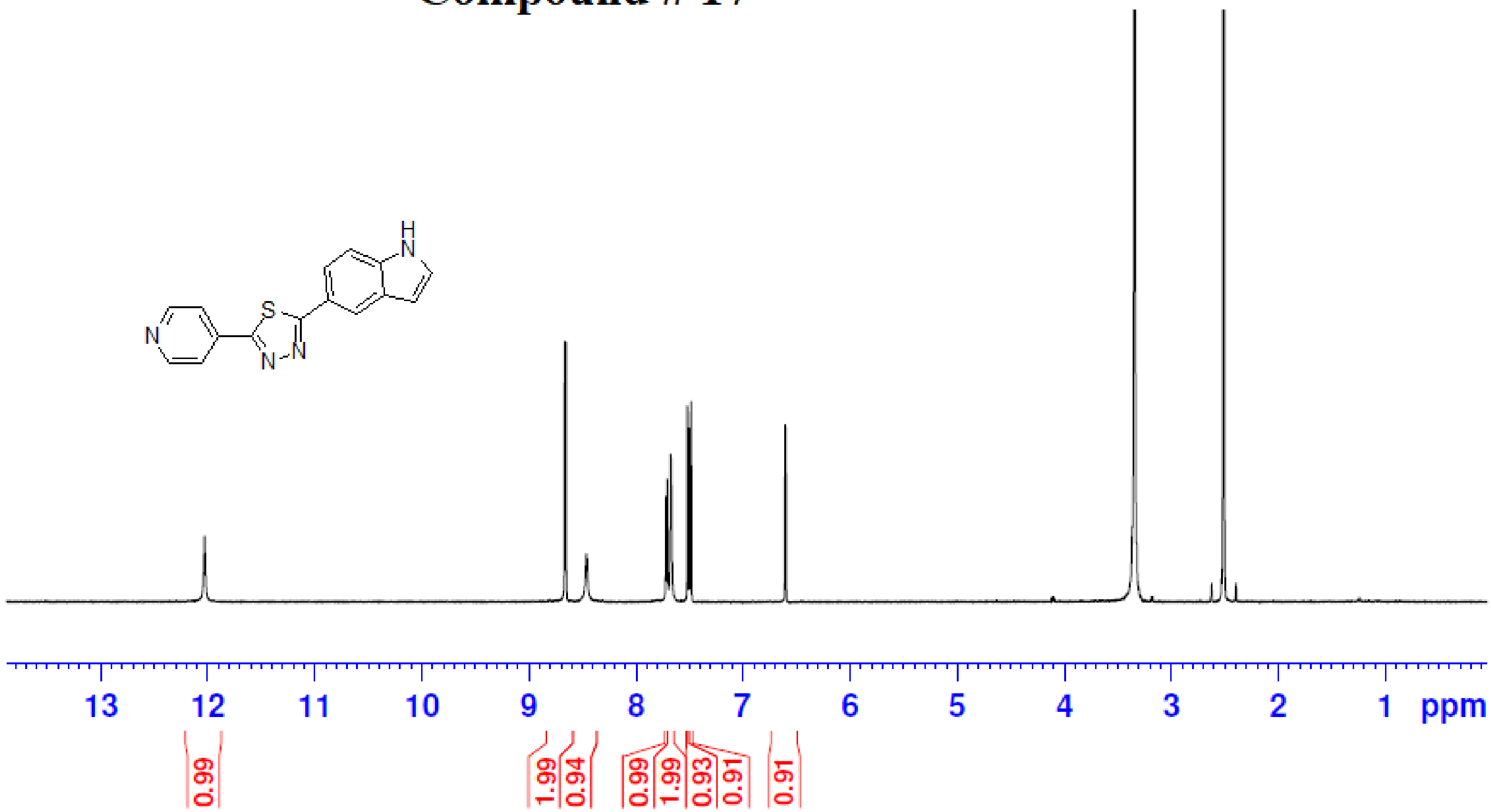
N
S
N
N
H
N
13
12
11
10
9
8
7
6
5
4
3
2
1
ppm
0.99
1.99
0.94
0.99
1.99
0.93
0.91
0.91

Compound # 18

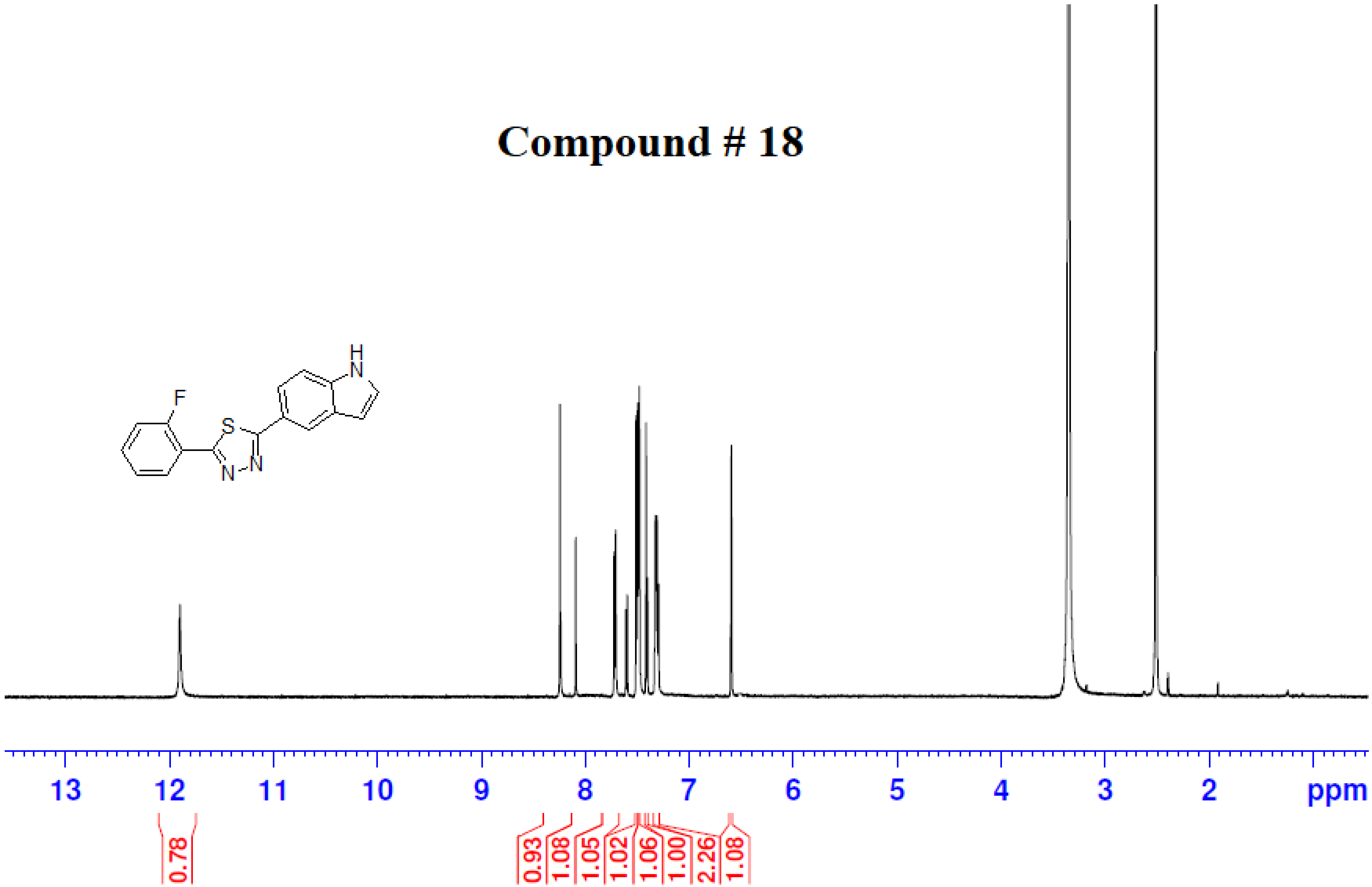

F
S
N
N
H
N
13
12
11
10
9
8
7
6
5
4
3
2
ppm
0.78
0.93
1.08
1.05
1.02
1.06
1.00
2.26
1.08

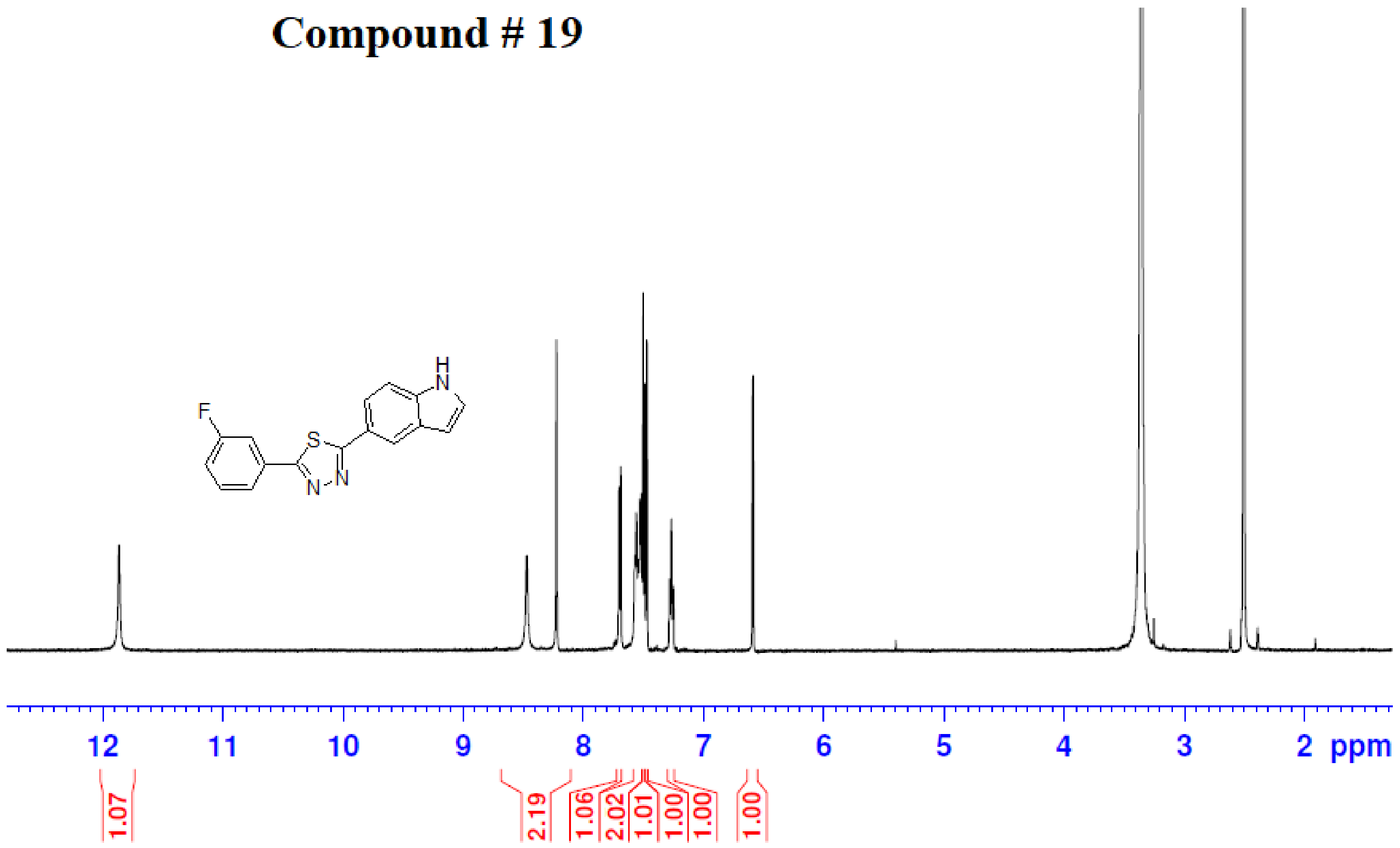
Compound # 19
F
S
N
N
H
N
12
11
10
9
8
7
6
5
4
3
2 ppm
1.07
2.19
1.06
2.02
1.01
1.00
1.00
1.00

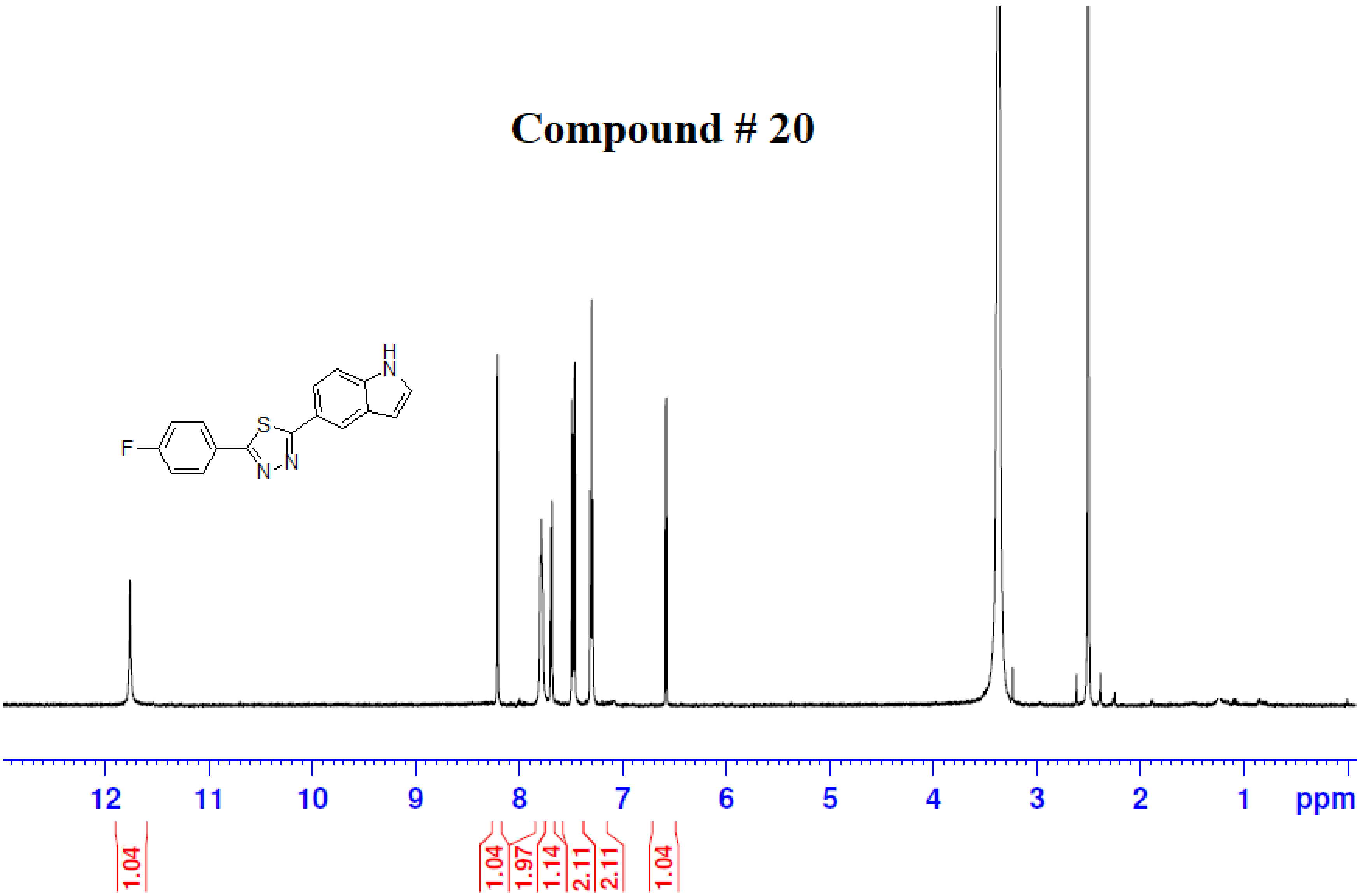

Compound # 20
F
S
N
N
H
N
12
11
10
9
8
7
6
5
4
3
2
1
ppm
1.04
1.04
1.97
1.14
2.11
2.11
1.04

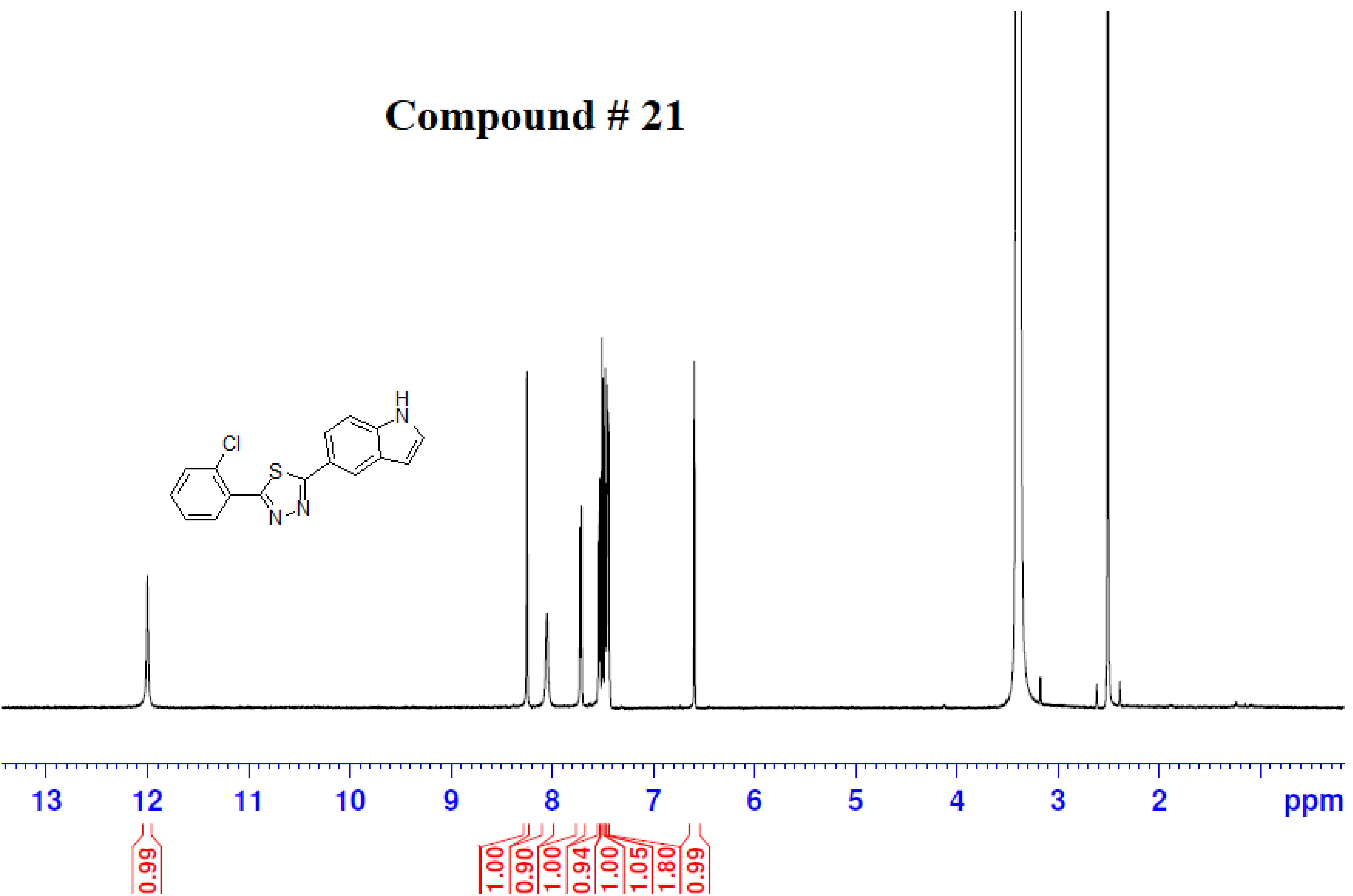
Compound # 21
Cl
S
N
N
H
N
13
12
11
10
9
8
7
6
5
4
3
2
ppm
0.99
1.00
0.90
1.00
0.94
1.00
1.05
1.80
0.99

Compound # 22

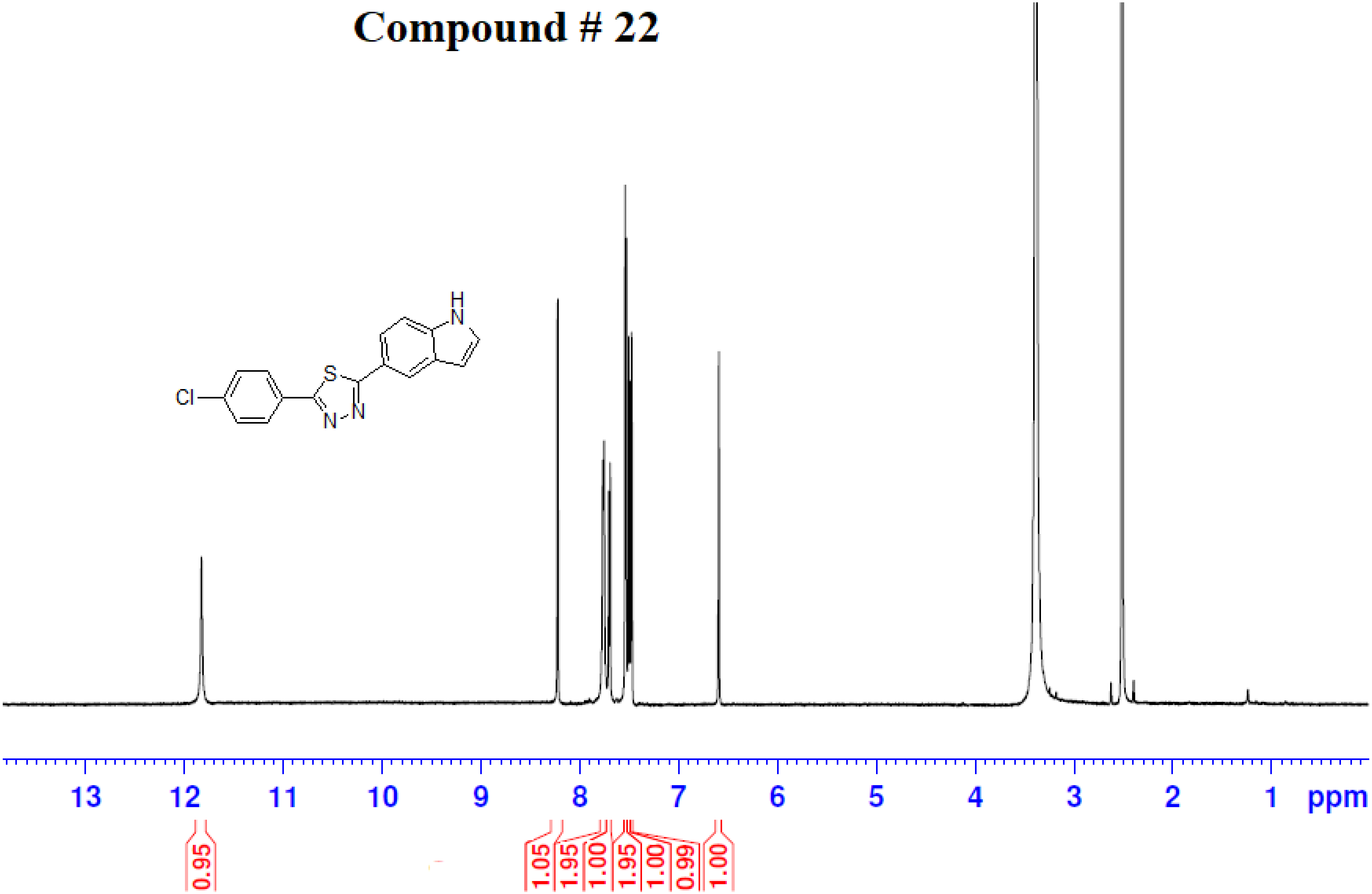

Cl
S
N
N
H
N
13 12 11 10 9 8 7 6 5 4 3 2 1 ppm
0.95
1.05
1.95
1.00
1.95
1.00
0.99
1.00
